# Quantitative, titratable and high-throughput reporter assays to measure DNA double strand break repair activity in cells

**DOI:** 10.1093/nar/gkad1196

**Published:** 2023-12-18

**Authors:** Eeson Rajendra, Diego Grande, Bethany Mason, Daniela Di Marcantonio, Lucy Armstrong, Graeme Hewitt, Elias Elinati, Alessandro Galbiati, Simon J Boulton, Robert A Heald, Graeme C M Smith, Helen M R Robinson

**Affiliations:** Artios Pharma Ltd, Babraham Research Campus, Cambridge CB22 3FH, UK; Artios Pharma Ltd, Babraham Research Campus, Cambridge CB22 3FH, UK; Artios Pharma Ltd, Babraham Research Campus, Cambridge CB22 3FH, UK; Artios Pharma Ltd, Babraham Research Campus, Cambridge CB22 3FH, UK; Artios Pharma Ltd, Babraham Research Campus, Cambridge CB22 3FH, UK; The Francis Crick Institute, London NW1 1AT, UK; Artios Pharma Ltd, Babraham Research Campus, Cambridge CB22 3FH, UK; Artios Pharma Ltd, Babraham Research Campus, Cambridge CB22 3FH, UK; Artios Pharma Ltd, Babraham Research Campus, Cambridge CB22 3FH, UK; The Francis Crick Institute, London NW1 1AT, UK; Artios Pharma Ltd, Babraham Research Campus, Cambridge CB22 3FH, UK; Artios Pharma Ltd, Babraham Research Campus, Cambridge CB22 3FH, UK; Artios Pharma Ltd, Babraham Research Campus, Cambridge CB22 3FH, UK

## Abstract

Repair of DNA damage is essential for the maintenance of genome stability and cell viability. DNA double strand breaks (DSBs) constitute a toxic class of DNA lesion and multiple cellular pathways exist to mediate their repair. Robust and titratable assays of cellular DSB repair (DSBR) are important to functionally interrogate the integrity and efficiency of these mechanisms in disease models as well as in response to genetic or pharmacological perturbations. Several variants of DSBR reporters are available, however these are often limited by throughput or restricted to specific cellular models. Here, we describe the generation and validation of a suite of extrachromosomal reporter assays that can efficiently measure the major DSBR pathways of homologous recombination (HR), classical nonhomologous end joining (cNHEJ), microhomology-mediated end joining (MMEJ) and single strand annealing (SSA). We demonstrate that these assays can be adapted to a high-throughput screening format and that they are sensitive to pharmacological modulation, thus providing mechanistic and quantitative insights into compound potency, selectivity, and on-target specificity. We propose that these reporter assays can serve as tools to dissect the interplay of DSBR pathway networks in cells and will have broad implications for studies of DSBR mechanisms in basic research and drug discovery.

## Graphical Abstract

**Graphical Abstract:**
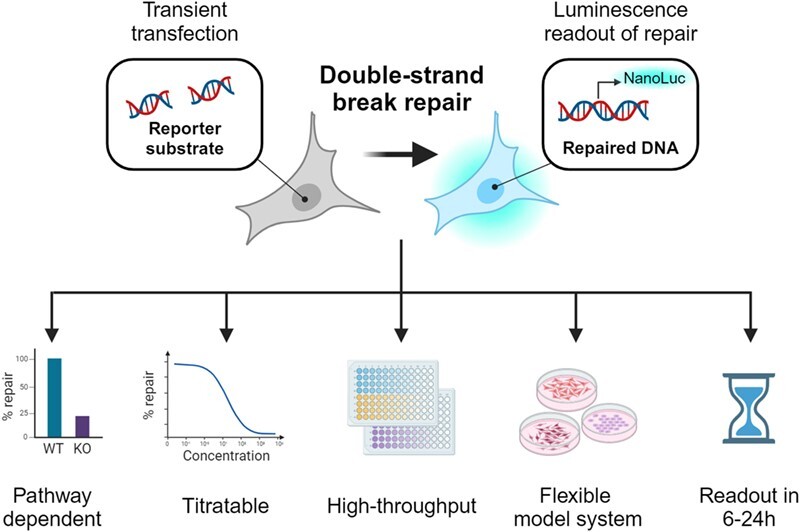

## Introduction

The repair of DNA double strand breaks (DSBs) provides essential protection from mutations, gross chromosomal rearrangements and chromosome loss, thereby preventing genome instability and potentially cell death (1). The importance of DSB repair (DSBR) is underscored by its critical role in healthy human physiology, its mis-regulation in a variety of disease states such as cancer and the consequent potential for targeted therapeutic exploitation.

There are several cellular DSBR pathways (2), which can be broadly categorised into homology-directed repair (HDR) mechanisms (homologous recombination (HR), microhomology-mediated end joining (MMEJ) and single strand annealing (SSA)) and homology-independent mechanisms that directly ligate broken ends such as classical nonhomologous end joining (cNHEJ).

All HDR mechanisms are initiated by the critical step of DNA end resection, which generates 3′ single strand DNA (ssDNA) ends by nucleolytic degradation. Downstream processing and repair occurs through distinct pathways such as HR, MMEJ and SSA. In HR, resection generates 3′ ssDNA tails flanking the DSB, which are initially coated by the ssDNA binding protein RPA. The HR mediator BRCA2 then nucleates and stabilises a RAD51 nucleoprotein filament that displaces the RPA. The RAD51 filament catalyses the formation of a D-loop structure by strand invasion of the broken molecule into a homologous donor sequence, which serves as a template for DNA synthesis from the invading strand. Subsequent intermediates are processed leading to gene conversion products, break-induced replication (BIR) or synthesis-dependent strand annealing (SDSA) products (3).

In MMEJ, resection exposes 3′ ssDNA overhangs flanking the DSB. Short microhomologies, typically 2–8 bp in length, are then annealed together to synapse the break ends before cleavage of the non-homologous 3′-ssDNA flaps occurs if the microhomologies are not terminal. The annealed, microhomologous bases then act as a platform for DNA polymerisation to fill in the remaining gaps, which may trigger the generation of a 5′-flap by polymerase-mediated strand displacement. Upon cleavage of the 5′-flaps, the nicked backbones are re-ligated to fully restore the duplex. The central player in this reaction is DNA polymerase theta (Polθ), a multifunctional enzyme, which comprises an N-terminal helicase domain and a C-terminal polymerase domain (4–7). By virtue of these domains, Polθ has the unique capacity to mediate both annealing of microhomologies and the subsequent polymerisation step. The helicase domain mediates the removal of RPA protein from ssDNA ends and stimulates microhomology annealing, whereas the polymerase domain extends the ssDNA ends from the microhomology and fills the remaining gaps (8–11). Both helicase and polymerase activities are essential for MMEJ *in vivo*.

In comparison to MMEJ, SSA involves both longer range resection and the exposure of extended homologous sequences flanking the DSB (12). The annealing of the homologies is mediated by the RAD52 and HELQ enzymes, which both possess intrinsic annealing activity on RPA coated ssDNA (13). SSA often leads to large deletions between the homologies.

In contrast to HDR mechanisms, cNHEJ involves the partial processing and ligation of DSBs in a sequence-independent manner when no homologous template is available. Ends can be blunt or have minimal overhangs. Many proteins have been described to be involved at different stages of the reaction, including Ku70/Ku80 and DNA-PKcs that are required for end recognition, Artemis and PNKP that are required if end-processing is needed as well as XLF, XRCC4 and Ligase IV that are required for ligation (14).

The importance of DSBR mechanisms in human physiology and their potential for therapeutic exploitation (15–17) necessitates titratable and sensitive assays that measure their occurrence, integrity and efficiency in physiological systems. Many approaches have been developed to measure cellular DSBR using reporter assay systems (18). These are often stably integrated in the genome of a specific cell line (chromosomal reporters) and the functional reconstitution of a reporter gene such as GFP by a specific DNA repair mechanism serves as a surrogate readout for the integrity of the repair pathway in cells (19). Alternatively, repair can also be monitored using substrates introduced by transient transfection (extrachromosomal assays), but these are less common.

A major advantage of chromosomal systems is that a repair event can be observed in its most physiological context i.e. on chromatinised DNA and understood in the context of relevant regulatory signalling cues as well as the accessory factors that constitute the cellular DNA damage response (DDR). However, existing chromosomal reporter assays have several limitations which preclude their expanded use in drug discovery settings, where functional cellular assays that are sensitive to pathway inhibition by small molecules can be critical to cellular screening cascades. Although excellent for characterising DNA repair in many settings, the requirement for high signal-to-noise ratios, exquisite sensitivity, robustness and reproducibility for quantitative high-throughput analyses limit their utility in small molecule drug discovery. Many of these assays also take up to 96 h to run which can affect interpretation of results if, for example, an inhibitor affects cell death and/or leads to gross cell cycle changes within this timeframe. From a practical perspective, sample handling for flow cytometry-based readouts can also limit throughput and analyses may be restricted to a certain cell line model in which the reporter has been genomically integrated.

As an alternative approach to chromosomal systems, the extent to which extrachromosomal DSBR reporter systems can robustly detect genetic and pharmacological modulation of factors has not been fully explored, nor whether the major DSBR mechanisms can function on extrachromosomal substrates. We have recently reported the generation of an extrachromosomal MMEJ reporter assay, which has been used to identify the first highly specific and selective small molecule inhibitors of the polymerase activity of Polθ (20,21). Here we describe the development and validation of this assay as well as a further suite of validated reporter assays for the other major DSBR pathways (cNHEJ, HR and SSA) based on the same format. These reporter assays circumvent many of the issues associated with other DNA repair assay formats that make them suitable for use as functional cell-based assays to measure the genetic and pharmacological modulation of DSBR proficiency in cells. These assays are easy to use, can be employed in any transfectable cell line and use a plate-based readout amenable to high throughput studies essential for small molecule drug discovery.

## Materials and methods

### Cloning

#### Resection-independent MMEJ reporter

The core substrate vector, generated by gene synthesis (GeneArt), comprised a CMV promoter downstream of the nanoluciferase-PEST gene and SV40 poly A terminator. The sequences were derived, and rearranged, from pNL3.2CMV (Promega) with the incorporation of silent nucleotide substitutions introducing a XhoI restriction site into the nanoluciferase coding region and eliminating a second HindIII site from the multiple cloning site. The PEST domain was removed by excision of a XmnI/XbaI fragment and replacement by a duplex DNA molecule generated from annealed oligonucleotides. A region of the vector was excised by restriction digest with XhoI and HindIII enzymes (NEB), followed by dephosphorylation with Antarctic Phosphatase (NEB). The core substrate fragment was separated from the vector backbone by agarose gel electrophoresis and purified by gel extraction (Qiagen).

ssDNA/dsDNA caps were annealed and ligated onto the substrate fragment in a 6:6:1 ratio (left cap: right cap: core) at 16°C overnight using T4 DNA ligase (NEB). Each cap comprised two functional ends: a 4-nt ‘sticky end’ suitable for specific ligation to a complementary end of the core substrate fragment (left = XhoI, right = HindIII) and a 45-nt 3′-ssDNA overhang comprising a terminal 4-nt microhomology to the other cap (5′-ATGG (right)/5′-CCAT (left)). The ligation reaction was separated from unligated excess caps by either a further round of agarose gel electrophoresis and gel extraction or purification using AMPure XP Beads (Beckman Coulter) to generate the reporter substrate.

Oligonucleotides used in this study are described in Supplementary Table 1.

MMEJ and NHEJ control plasmids, mimicking the products of MMEJ or NHEJ-mediated repair respectively, were generated by gene synthesis (GeneArt). The sequence coding for the PEST domain was removed using the same method as described above.

#### Blunt end NHEJ reporter

The core substrate vector, generated by gene synthesis (GeneWiz), comprised the C-terminal region of the nanoluciferase gene upstream of a SV40 poly A terminator, followed by a CMV promoter upstream of the N-terminal region of the nanoluciferase gene. The sequences were derived and rearranged from pNL3.2CMV (Promega) with the incorporation of silent nucleotide substitutions introducing an EcoRV restriction site into the nanoluciferase coding region. A region of the vector was excised by restriction digest with EcoRV and the blunt end fragment constituting the reporter substrate was separated from the vector backbone by agarose gel electrophoresis and purified by gel extraction (Qiagen).

#### Resection-dependent MMEJ, non-cohesive end NHEJ, long and short template HR and SSA reporters

Plasmids encoding the reporter substrates were generated by gene synthesis (GeneWiz) and subcloned into pUC57 (Kan). Vectors were digested with I-SceI (ThermoFisher) or HindIII (NEB) and purified using either a PCR purification kit (Qiagen) or AMPure XP Beads (Beckman Coulter).

All reporter substrates presented in the manuscript use a nanoluciferase-based reporter gene, which encodes NanoLuciferase (NanoLuc) protein. Promega is the source of the NanoLuc® technology and the modified NanoLuc® polynucleotides. The modified NanoLuc® polynucleotide sequences used in this paper, which encode the NanoLuc® reporter protein upon repair, differ from the canonical sequence by up to four nucleotide substitutions (T72C, A75G, G114C, C171T). These substitutions are functionally silent. Artios Pharma was authorised by Promega to generate the modified NanoLuc® polynucleotides.

#### Transfection control plasmids

Firefly luciferase was used as a transfection/normalisation control and its expression plasmid co-transfected alongside nanoluciferase-encoding plasmids and extrachromosomal reporters unless indicated otherwise.

CMV-Firefly was generated by gene synthesis (GeneWiz) and subcloned into pcDNA3.1 (+) (Invitrogen) as an EcoRI-EcoRV fragment. pGL4.53 (Promega) encodes Firefly under a PGK promoter and was used in Figures S1 and S8.

I-SceI-T2A-Firefly, encoding a polypeptide comprising I-SceI and Firefly luciferase separated by an intracellular T2A cleavage site, was generated by gene synthesis (GeneWiz) and cloned into pcDNA3.1(+) as a NheI–XbaI fragment. This plasmid, used in Supplementary Figure S3B, was co-transfected with an undigested plasmid encoding a reporter for assays in which I-SceI cleavage to generate the DSB was performed intracellularly.

### Cell culture

Cell lines were cultured in recommended media (PAN-Biotech or Gibco) supplemented with 10% foetal bovine serum (PAN-Biotech) under normal growth conditions (37°C, 5% CO_2_), and passaged at 70–80% confluency. Cell lines used in this study are described in Supplementary Table 2. Induction of GFP-Polθ expression in U-2 OS Flp-In T-REx GFP-POLQ cells was performed with doxycycline (Sigma-Aldrich) for 24 h prior to assays.

### Reporter assays

Cells were harvested by trypsinisation, washed with PBS, resuspended in fresh culture medium, and counted. Transfection with reporter substrates was performed as outlined below.

#### Lipofection

Transfection complexes were prepared by transferring nanoluciferase DNA substrate and pcDNA3.1(+)-Firefly[CMV/luc2] plasmid generated by gene synthesis (GeneWiz) into jetPRIME buffer (Polyplus) at a ratio of 100–1000 ng nanoluciferase DNA substrate: 400 ng Firefly plasmid: 80 μL jetPRIME buffer for every 200 000 cells. jetPRIME reagent was later added at a ratio of 2 μl per 1 μg DNA, each tube was vortexed for 5 s and incubated at room temperature for 10 min. Transfection complexes were added to cells while in suspension just before seeding. 10 000–20 000 cells were seeded per well in a white 96-well microplate (Costar 3610 or Porvair 214006) and incubated for 16–24 h at 37°C.

For U-2 OS Flp-In T-REx GFP-POLQ cells, transfection complexes were prepared in Opti-MEM buffer (ThermoFisher) and transfected using FuGENE HD reagent (Promega) at a ratio of 3 μl per 1 μg DNA.

Unless indicated otherwise, lipofection was used to perform reporter assays.

#### Nucleofection

Cells were centrifuged at 90 × g for ten minutes and resuspended in supplemented SE or SF nucleofection solution (Lonza), as recommended by the manufacturer. The nanoluciferase DNA substrate and Firefly luciferase plasmid (Promega) were added at a ratio of 20 μl SE/SF: 100–400 ng nanoluciferase substrate: 400 ng Firefly plasmid: 100 000–200 000 cells. Cells were transferred to a cuvette, electroporated using the recommended programme by the manufacturer on the 4D nucleofector X unit (Lonza) and recovered into fresh media to a final density of 125 000–250 000 cells/ml. 10 000–20 000 cells (80 μl of suspension) were seeded per well in a white 96-well microplate (Costar 3610 or Porvair 214006) and incubated for 16–48 h at 37°C.

To assess the function of wild-type and mutant Polθ in MMEJ, transient overexpression of GFP-POLQ constructs was performed in U-2 OS POLQ^−/−^ cells by nucleofection with 5 μg pcDNA5/FRT/TO-GFP-POLQ plasmid per 10^6^ cells 24 h prior to nucleofection of the MMEJ reporter substrate.

To assess titratable modulation of DNA repair, compounds were dispensed using the Tecan D300e digital dispenser to generate a multipoint dose response curve, with a backfilling step included to equalise the final DMSO (Sigma-Aldrich) concentration. Cells were transfected with the reporter substrate and plated to wells containing compound.

Firefly and NanoLuc levels were detected using the Nano-Glo® Dual-Luciferase® Reporter Assay system (Promega) as per the manufacturer's instructions, and luminescence was measured with a CLARIOstar plate reader (BMG Labtech), using the manufacturer's protocols ‘Firefly’ and ‘NanoLuciferase’. In each well, the NanoLuc signal was normalised to the Firefly signal, which served as a measure of both cell density and transfection efficiency.

#### siRNA transfection

Cells were washed in PBS and transfected with indicated siRNAs at 10–20 nM final concentration using Lipofectamine RNAiMAX (Invitrogen) according to manufacturer's instructions. After 72 h, cells were transfected with siRNA and the indicated reporter substrate (s) for a further 24 h, or harvested for western blot analysis. siRNAs used in this study are described in Supplementary Table 3.

### Western blot

Cells were washed in PBS, lysed directly in RIPA buffer (ThermoFisher) and protein extracts were quantitated using the Bicinchoninic acid assay (ThermoFisher) against a BSA standard curve. Extracts were made up in 4× NuPAGE LDS sample loading buffer (Invitrogen) supplemented with 100 mM dithiothreitol (Sigma-Aldrich), and incubated at 95°C for 10 min. Lysates (50–100 μg) were resolved by SDS-polyacrylamide gel electrophoresis (SDS-PAGE) on NuPAGE 3–8% Tris–acetate gels (Invitrogen) in NuPAGE Tris–acetate running buffer (Invitrogen), or NuPAGE 4–12% Bis–Tris gels (Invitrogen) in NuPAGE MOPS SDS running buffer (Invitrogen). Gels were wet-transferred in 1× NuPAGE Transfer Buffer (Invitrogen), 20% ethanol and 0.05% SDS to nitrocellulose membranes (Millipore). 5% BSA/Tris-buffered saline + 0.01% Tween-20 (TBST) or 5% milk/TBST was used for blocking and incubation steps. Membranes were probed overnight at 4°C with indicated antibodies. The membrane was washed thrice for 5 min with TBST and incubated with HRP- or fluorescent dye-conjugated secondary antibodies for 1 h at room temperature. After four 5 min washes with TBST, HRP signals were detected with ECL detection reagent (BioRad) and imaged on an Amersham Imager 600RGB and fluorescence was imaged directly on a LI-COR Odyssey M Imager. Antibodies used in this study are described in Supplementary Table 4.

### Antibody generation

A rabbit polyclonal antibody against human Polθ was generated by Cambridge Research Biochemicals by immunisation with a bacterially produced GST-fusion protein encoding residues 1290–1389 generated by Peak Proteins.

### Cell line generation

U-2 OS Flp-In T-REx GFP-POLQ cells were generated as follows: U-2 OS cells were acquired from ATCC and transfected with pFRT/lacZeo (Invitrogen). Single clones were selected based on resistance to Zeocin 400 μg/ml (Invitrogen) and by performing a β-galactosidase assay (Thermo) according to manufacturer's specifications. Single integration of the FRT site was confirmed by Southern blot. The U-2 OS Flp-In clone was then transfected with pcDNA6/TR (Invitrogen) and a pool of cells resistant to Zeocin 100μg/ml and Blasticidin S HCl 8μg/ml (Invitrogen) was selected. The U-2 OS Flp-In T-REx pool was then co-transfected with the Flp-Recombinase expression vector pOG44 (Invitrogen) and pcDNA5/FRT/TO-GFP-POLQ (custom made by GeneWiz). U-2 OS Flp-In T-REx GFP-POLQ clone 4 was isolated based on resistance to Blasticidin 8 μg/ml and Hygromycin 200 μg/ml.

U-2 OS POLQ^−/−^ cells were generated by Synthego and knock-out status confirmed by western blotting.

eHAP1 POLQ ^(–)^ cells were previously described (22).

### Rad51 foci formation assay

DLD-1 cells were seeded at a concentration of 10 000 cells/well in collagen-coated 96-well plates (Perkin Elmer 6055700) and grown overnight. Cells were treated with increasing concentrations of CAM833 for one hour prior to dosing with 5 Gy ionizing irradiation (Faxitron CellRad X-Ray System—2328A50201). Six hours post-irradiation, medium was removed, and cells were washed twice with PBS before pre-extraction with CSK buffer (10 mM PIPES pH 6.8 (Sigma Aldrich), 100 mM NaCl (VWR), 300 mM Sucrose (Sigma Aldrich), 1.5 mM MgCl_2_ (VWR), 5 mM EDTA (Sigma Aldrich), 0.5% Triton X-100 (Sigma Aldrich)) for 3 min at room temperature. Cells were fixed in 4% (w/v) paraformaldehyde (PFA) in PBS for 10 min at room temperature (RT), washed twice with PBS, and blocked with 0.5% (w/v) BSA, 0.5% (v/v) Triton X-100 in PBS (Blocking Buffer, BB) for 1 h at RT. Cells were then incubated with anti-RAD51 primary antibody diluted in blocking buffer at 4°C overnight. Cells were washed twice with PBS + 0.1% Triton X-100 and incubated with Alexa Fluor 488-conjugated rabbit secondary antibody (Invitrogen, A11034) and 1 μg/ml DAPI in BB for 1 h at room temperature. Cells were then washed twice with PBS + 0.1% Triton X-100 and imaged in 100 μl of PBS. Immunofluorescence images were acquired using a 20× hNA Air objective with the Operetta CLS High-Content Imaging System (Perkin Elmer). Quantification of the number of RAD51 foci was performed using the Harmony V4.9 image analysis software.

### Compounds

Compounds used in this study are described in Supplementary Table 5.

### Data analysis

Data were collated in Microsoft Excel and GraphPad Prism analytical software was used to generate graphs and to perform curve fitting and statistical analysis. Statistical significance was determined by a Student's *t*-test (two-tailed, unpaired) or two-way ANOVA as indicated in the figure legends; ‘n.s.’ = not significant (*P*> 0.05), *P ≤ 0.05, **P ≤ 0.01, ***P ≤ 0.001, ****P ≤ 0.0001. Software used in this study is described in Supplementary Table 6.

## Results

### Generation and validation of MMEJ reporter assay substrates

We set out to build a suite of extrachromosomal DSBR reporter assays for measuring the cellular potency, selectivity and kinetics of targeted DNA repair inhibitors in a robust and high-throughput manner.

The development of an MMEJ reporter assay is of particular interest as targeting its key mediator, Polθ, has been shown to be a promising synthetic lethal target in HR-deficient (HRD) cancers (23,24). Measurement of MMEJ-mediated repair of an extrachromosomal DNA substrate by Polθ has been previously reported (10). In that study, the repair substrate comprised a central dsDNA region with flanking ssDNA overhangs terminating in 4 nucleotide microhomologies. Polθ-mediated MMEJ aligned these microhomologies and performed fill-in synthesis to generate a dsDNA product which could be distinguished from other end joining products generated by cNHEJ by altered electrophoretic mobility and quantitative PCR analysis of the substrate once recovered from cells. Although suitable for the characterisation of MMEJ repair in a low throughput manner, the scope for using this assay in a high-throughput screening setting was limited by scalability of the PCR format and amenability to compound screening. We hypothesised that these limitations could be circumvented by adapting the DNA substrate to function as a sensitive cellular reporter assay to detect MMEJ-mediated repair.

**Figure 1.**
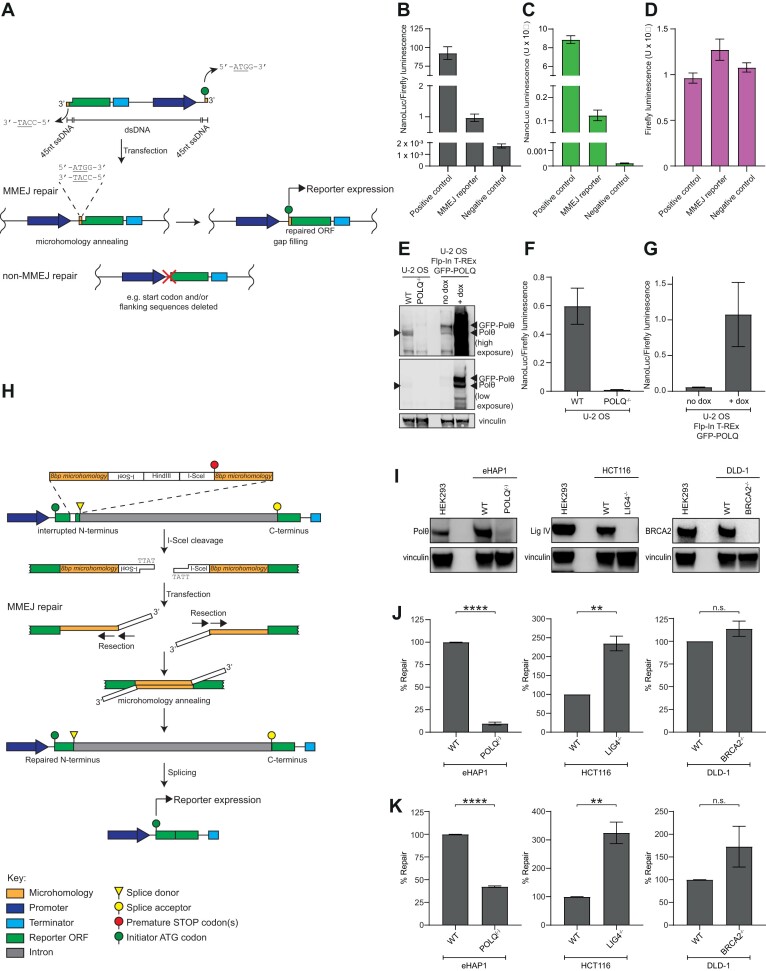
Schematic of extrachromosomal MMEJ reporter assay substrates and their genetic validation. (**A**) Schematic representation of the resection-independent MMEJ reporter substrate. Upon transfection, cellular MMEJ-mediated repair via terminal microhomologies on ssDNA overhangs reconstitutes a functional reporter ORF expressing NanoLuc. (B–D) Repair of the resection-independent MMEJ reporter generates a NanoLuc luminescence signal. HEK293 cells were transfected with the resection-independent MMEJ reporter substrate, a plasmid expressing an intact nanoluciferase ORF representing correct MMEJ repair (positive control), or a plasmid expressing an ORF comprising nanoluciferase as if repaired by NHEJ at the ss/dsDNA junction after clipping of the ssDNA overhangs (negative control), alongside a Firefly control. (**B**) NanoLuc luminescence was normalised to Firefly luminescence to determine substrate repair 24 h post-transfection for the reporter alongside signal from the control plasmids. The positive control and the (repaired) reporter substrate generated robust repair signals. (**C**) NanoLuc signals alone show the positive control and the (repaired) reporter substrate generated robust NanoLuc signals. (**D**) The Firefly signal, acting as a control, is stable under co-transfection with the positive control plasmid, MMEJ reporter substrate or negative control plasmid. Data represent mean ± SD of 8 technical replicates. (E–G) Repair of the resection-independent MMEJ reporter is dependent on Polθ. (**E**) Western blot confirming knockout of POLQ in U-2 OS and the doxycycline-induced overexpression of GFP-Polθ in a U-2 OS Flp-In T-REx cell line. (**F**) Genetic ablation of POLQ eliminates repair of the resection-independent MMEJ reporter substrate. (**G**) Overexpression of Polθ increases repair of the resection-independent MMEJ reporter substrate. Data represent mean ± SEM of 2 biological replicates each averaging 6 technical repeats. (**H**) Schematic representation of the resection-dependent MMEJ reporter substrate. I-SceI cleavage generates a non-cohesive DSB in an interrupted N-terminal fragment of the reporter gene. The break is flanked by 8bp microhomologies embedded in dsDNA regions. Upon transfection, end resection at the break is required to expose the microhomologies and permit MMEJ-mediated repair of the N-terminal fragment. Upon repair, the N- and C-terminal portions of the reporter gene are spliced together reconstituting an ORF expressing NanoLuc. (I, J) Genetic ablation of POLQ reduces MMEJ repair. (**I**) Western blot of isogenic cell line pairs confirming knockout of BRCA2, POLQ and LIG4 in DLD-1, eHAP1 and HCT116 cells. HEK293 cells are shown for comparison. (**J**) POLQ^(–)^ cells show defective repair of the resection-independent MMEJ reporter shown in (A). (**K**) POLQ^(–)^ cells show defective repair of the resection-dependent MMEJ reporter shown in (H). NanoLuc luminescence was normalised to Firefly luminescence to determine substrate repair 24h post-transfection. In (J) and (K), % repair of knockout cells is expressed relative to their respective parental WT cell line. Data represent mean ± SEM of 3 biological replicates, each averaging 8 technical replicates. Significance was determined by a Student's t-test (two-tailed, unpaired).

The reporter gene we opted to use for these assays is nanoluciferase, which encodes NanoLuc luciferase. It was selected based on its biophysical properties (brightness and stability), its rapid maturation, as well as its low molecular weight, which reduces the size of the transfected reporter substrate (25). The layout of the reporter is shown in Figure 1A. The detailed generation of this substrate is described in the Methods. The arrangement of the reporter substrate places the nanoluciferase open reading frame (ORF) and terminator upstream of a CMV promoter. The termini of both ends of the substrate are in a 45 nucleotide ssDNA context generated by ligation of short oligonucleotides to a dsDNA core region. These ssDNA overhangs containing a 4 nucleotide microhomology (5′-ATGG-3′/3′-TACC-5′) and encompassing the ATG start codon are ligated at each terminus. Repair of the substrate through MMEJ both restores the ATG and places the ORF downstream of the promoter allowing expression of the nanoluciferase gene. As this reporter represents an MMEJ repair substrate downstream of DSB end resection, we termed this substrate the ‘resection-independent’ MMEJ reporter.

The resection-independent MMEJ reporter assay was performed by co-transfecting the linear reporter substrate into cells with a circular plasmid encoding Firefly luciferase, which served as a transfection control. After 24 h, the generation of both the NanoLuc and Firefly luciferase was determined on a plate reader and the extent of repair defined by the ratio of the signals from the MMEJ substrate (NanoLuc) and the transfection control (Firefly).

To establish that the signal we observed from the resection-independent MMEJ reporter assay was specific, we transfected HEK293 cells with plasmids expressing nanoluciferase ORFs mimicking the expected products of either MMEJ-mediated repair (positive control) or a cNHEJ-mediated repair (negative control) event after the ssDNA overhangs have been cleaved. Only the MMEJ reporter substrate and the model MMEJ product generated a repair signal (Figure 1B). Evaluation of the individual NanoLuc (Figure 1C) and Firefly signals (Figure 1D) revealed that the detectable signal is a consequence of the MMEJ product encoding NanoLuc, confirming that the resection-independent MMEJ reporter specifically measures MMEJ and not cNHEJ.

Dependence on Polθ was confirmed using a U-2 OS cell line in which the POLQ gene was deleted and a U-2 OS cell line in which overexpression of GFP-Polθ could be induced, as confirmed by Western blot (Figure 1E). We observed that Polθ loss ablated MMEJ (Figure 1F), whereas induction of GFP-Polθ strongly increased MMEJ above the basal levels repaired by endogenous Polθ (Figure 1G). Together these data confirm that Polθ-mediated MMEJ is responsible for the repair signal in the resection-independent MMEJ reporter assay.

We additionally generated a ‘resection-dependent’ MMEJ reporter as shown in Figure 1H. The N- and C-terminal exons of the ORF are interrupted by sequence including a stop codon and thus cannot express functional NanoLuc. The stop codon is flanked by sequence encoding 8 bp microhomologies, with tandem I-SceI sites embedded between the two microhomologies, upstream of the stop. The DSB is generated upstream of the stop codon by *in vitro* cleavage by I-SceI, creating non-cohesive ends. Upon transfection, resection is necessary to reveal the microhomologies in their ssDNA context and allows MMEJ-mediated annealing and repair, deleting the stop codon and restoring an intact, functional nanoluciferase gene.

To validate the specificity of these MMEJ reporters, we assessed how the repair signal was impacted by genetic ablation of known components of three main DSBR pathways. Isogenic pairs of cell lines were selected with indicated deficiencies: POLQ (eHAP1 background), LIG4 (HCT116 background), and BRCA2 (DLD-1 background) representing MMEJ-deficient (MMEJD), NHEJ-deficient (NHEJD) and HRD contexts, respectively. Gene knockout (KO) was validated by Western blot (Figure 1I). In agreement with the data in U-2 OS cells (Figure 1F), ablation of POLQ reduced MMEJ-mediated repair of the resection-independent MMEJ reporter by ∼90% (Figure 1J), while deletion of BRCA2 or LIG4 did not reduce the reporter signal (Figure 1J). We also observed a ∼60% reduction in the MMEJ resection-dependent reporter signal upon KO of POLQ, whereas deletion of BRCA2 or LIG4 led to a 2- to 3-fold increase in the reporter signal, likely caused by the loss of competing DSBR pathways (Figure 1K) (26–28).

Collectively, these data indicate that these reporter assays specifically measure DNA repair by MMEJ and also highlight the ease of transferability of the assays between different cell lines e.g. DLD-1, eHAP1, HCT116 and U-2 OS.

### Generation and validation of cNHEJ reporter assay substrates

Having built and genetically validated MMEJ reporters, we deployed similar principles to design and generate extrachromosomal reporter assays for measuring activity of the other major DSBR pathways: NHEJ, HR and SSA. These can be used to both interrogate the specific impact on proficiency of these pathways by targeted genetic and pharmacological approaches as well as for profiling selectivity across these competing repair mechanisms.

**Figure 2.**
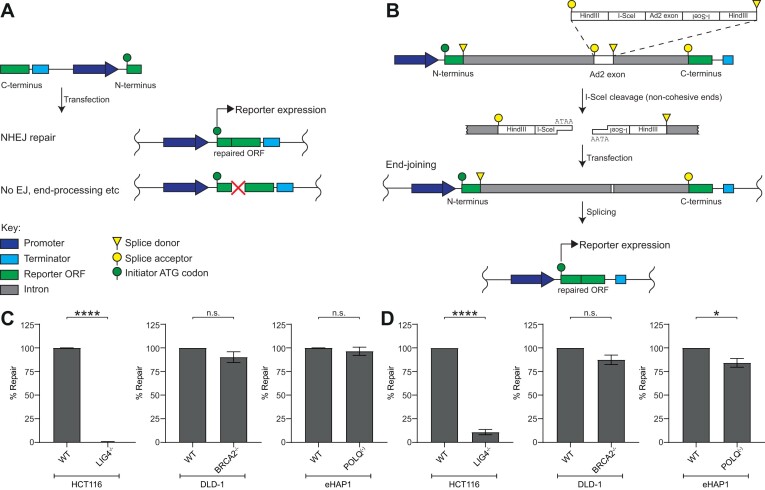
Schematic of extrachromosomal NHEJ reporter assay substrates and their genetic validation. (**A**) Schematic representation of the blunt-ended NHEJ reporter substrate. Upon transfection, NHEJ-mediated repair with no loss of information at the break reconstitutes a functional reporter ORF expressing NanoLuc. (**B**) Schematic representation of the non-blunt NHEJ reporter substrate. I-SceI cleavage excises a small exon from the parental plasmid and generates a non-cohesive DSB within an intronic region separating the N- and C-terminal portions of a reporter gene. Upon transfection, end processing followed by ligation repairs the break. Upon repair, the N- and C-terminal portions of the reporter gene are spliced together reconstituting an ORF expressing NanoLuc. (**C**) LIG4^−/−^ cells show complete ablation of repair of the blunt end NHEJ reporter shown in (A). (**D**) LIG4^−/−^ cells show almost complete ablation of repair of the non-cohesive NHEJ reporter shown in (B). NanoLuc luminescence was normalised to Firefly luminescence to determine substrate repair 24 h post-transfection. In (C) and (D), % repair of knockout cells is expressed relative to their respective parental WT cell line. Data represent mean ± SEM of 3 biological replicates, each averaging 8 technical replicates. Significance was determined by a Student's *t*-test (two-tailed, unpaired).

We generated two NHEJ reporters, with and without blunt ends. The layout of the blunt end NHEJ reporter substrate is shown in Figure 2A. The substrate arrangement, like the resection-independent MMEJ reporter, comprises an inverted expression cassette with the N-terminal portion of nanoluciferase downstream of a CMV promoter and the C-terminal portion upstream of the terminator sequence. The blunt-ended DSB is derived by cleavage of a silent EcoRV restriction site introduced within the nanoluciferase coding region. The functional nanoluciferase ORF is reconstituted when the unprocessed blunt ends are directly ligated, joining the N- and C-terminal regions of nanoluciferase without any end processing that could corrupt the sequences flanking the break.

The layout of the non-blunt NHEJ reporter substrate is shown in Figure 2B. The substrate arrangement is similar to a previously described reporter substrate (29). The engineered DSB is not blunt and depending on the choice of enzyme, the short overhangs can yield cohesive or non-cohesive ends. Cleavage by HindIII generates cohesive ends whereas I-SceI cleavage generates non-cohesive ends as the tandem sites are inverted. The non-cohesive end variant of the non-blunt NHEJ reporter has been used in this study. As these ends are non-complementary, they must be processed prior to ligation. In both cases, NHEJ-mediated repair generates an intact locus, joining the N- and C-terminal portions of nanoluciferase as exons that can be spliced together to generate a functional nanoluciferase ORF.

To ensure that the repair of these reporters is dependent on cNHEJ, we assessed their repair in LIG4, BRCA2 and POLQ KO cells compared to matched, isogenic cells wild-type for these genes. The repair of the blunt end NHEJ reporter (Figure 2C) and non-cohesive NHEJ (Figure 2D) reporters was significantly reduced in LIG4 KO cells, while loss of BRCA2 or POLQ did not affect the blunt end NHEJ reporter signal and reduced the non-cohesive NHEJ reporter signal by only ∼20%, neither of which are likely to generate an observable NHEJ deficiency. Similarly, we further demonstrated that repair was dependent on additional components of the cNHEJ core complex using isogenic XLF and XRCC4 KO cells (Figure S1).

The blunt end NHEJ reporter demonstrates exquisite genetic dependency on cNHEJ factors (Figures 2A, C and S1B). However, it should be noted that insertion/deletion (InDel)-generating cNHEJ events may not restore a functional NanoLuc ORF and thus be undetected in the assay.

### Generation and validation of HR reporter assay substrates

**Figure 3.**
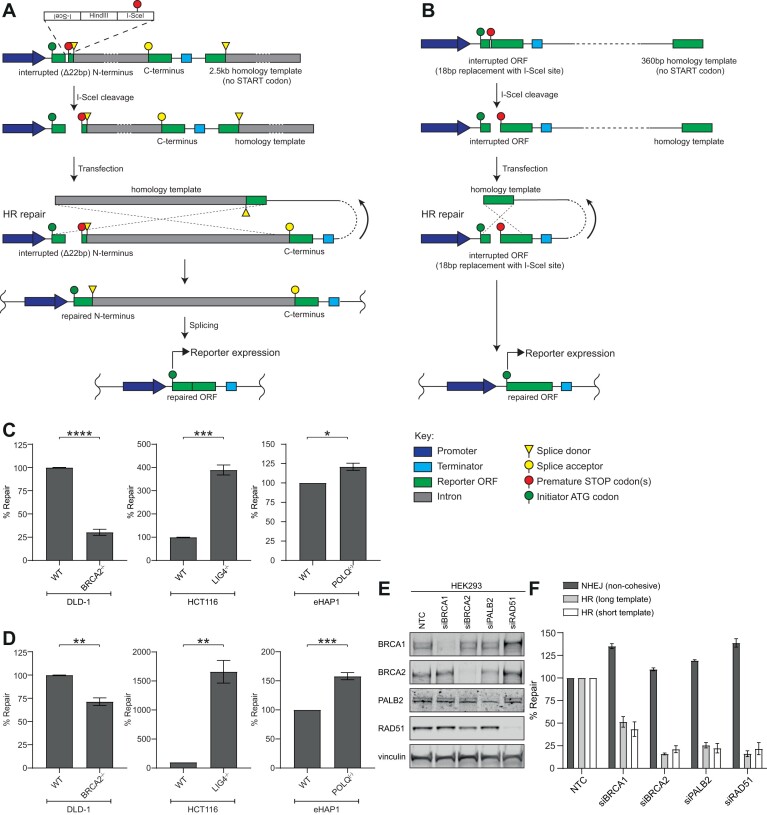
Schematic of extrachromosomal HR reporter assay substrates and their genetic validation. (**A**) Schematic representation of the long template HR reporter substrate. I-SceI cleavage generates a non-cohesive DSB in an interrupted N-terminal fragment of the reporter gene. Upon transfection, homology-directed repair of the break is templated by a large region (∼2.5 kb) containing the intact N-terminal fragment lacking a start codon. Upon repair, the N- and C-terminal portions of the reporter gene are spliced together reconstituting an ORF expressing NanoLuc. (**B**) Schematic representation of the short template HR reporter substrate. I-SceI cleavage generates a cohesive DSB in an interrupted N-terminal fragment of the reporter gene. Upon transfection, homology-directed repair of the break is templated by a smaller region (∼0.4 kb) containing the intact N-terminal fragment lacking a start codon. Upon repair, the reporter gene is reconstituted permitting expression of an ORF expressing NanoLuc. (**C**) BRCA2^−/−^ cells show defective repair of the HR reporter shown in (A) with a significant increase in repair observed in the LIG4^−/−^ cells. (**D**) BRCA2^−/−^ cells show defective repair of the HR reporter shown in (B). with a significant increase in repair observed in the LIG4^−/−^ cells. (**E**) Western blot confirming siRNA-mediated knockdown of BRCA1, BRCA2, PALB2 and RAD51 in HEK293 cells. NTC denotes non-targeting control. (**F**) After knockdown of BRCA1, BRCA2, PALB2 or RAD51, cells are unable to perform HR-mediated repair of the HR reporters shown in Figure 2 but do not affect the NHEJ reporter signal. All decreases in % repair seen for both HR reporters after knockdown of BRCA1, BRCA2, PALB2 or RAD51 are statistically significant (*P*< 0.05). NanoLuc luminescence was normalised to Firefly luminescence to determine substrate repair 24 h post-transfection. In (C) and (D), % repair of knockout cells is expressed relative to their respective parental WT cell line. Data represent mean ± SEM of three biological replicates, each averaging eight technical replicates. Significance was determined by a Student's *t*-test (two-tailed, unpaired).

We next designed two HR reporter assay substrates using direct repeat orientation homology templates, which have been widely used in chromosomal HR reporters (29,30). The long template homologous recombination substrate reporter outlined in Figure 3A is based on the HR construct layout in (29). It contains the nanoluciferase ORF separated into N- and C-terminal portions by an intron. The N-terminal portion contains a deletion of 22 bp of native sequence and the insertion of a short sequence encoding tandem I-SceI sites flanking a HindIII site and a stop codon and thus cannot express functional NanoLuc. Repair of the N-terminal exon must be templated by an approximately 2.5 kb downstream homology template, which lacks a functional start codon. *In vitro* cleavage by I-SceI digestion linearises the reporter plasmid and creates an engineered DSB with non-cohesive ends (Figure S3A). Upon transfection into cells, HDR mediates the generation of an intact N-terminal exon which can be spliced to the C-terminal portion to generate a functional reporter.

The short template HR reporter substrate in Figure 3B, has similarities to the chromosomal DR-GFP reporter construct as described in (30). The substrate comprises a complete reporter ORF, incorporating a sequence encoding a single I-SceI site and an in-frame stop codon, cleavage of which creates a DSB with cohesive ends. Removal of the in-frame stop codon and restoration of the full nanoluciferase ORF is dependent on HR repair using a short, approximately 360 bp downstream homology template, which lacks a functional start codon.

We validated these HR reporter assays by transfecting them into isogenic wild-type or KO cell lines for various DSBR genes. As shown in Figure 3C and D, transfection of the HR reporter substrates resulted in a NanoLuc signal. Consistent with this signal being due to repair by HR, we observed a significantly reduced signal in DLD-1 BRCA2 deficient (HRD) cells, although this effect was much more pronounced using the long template HR substrate (Figure 3C) than the short template HR substrate (∼75% reduction in the former compared to ∼30% reduction in the latter assay, Figure 3D). Consistently, we observed that siRNA-mediated knockdown of BRCA2 in DLD-1 cells prior to HR reporter transfection caused a reduction in the NanoLuc signal using both HR reporter substrates (∼80% reduction with the long template and ∼55% with the short template substrate, Figure S2). As expected, in NHEJ defective LIG4 KO cells, an increase in HR repair was observed above baseline, possibly indicating pathway compensation or reduced substrate sequestration when NHEJ is ablated (26,31,32). In MMEJ defective POLQ KO cells we observed a trend towards a slight increase in HR repair.

To further dissect the HR-dependent repair of the HR reporters we next sought to assess the impact of knockdown of a range of HR factors in HEK293 cells. Depletion of BRCA1, BRCA2, PALB2 and RAD51 by siRNA was confirmed by Western blot (Figure 3E). Loss of each factor similarly reduced repair of both HR reporters by ∼50–80% (Figure 3F). These data suggest that the reporters are sensitive to knockdown of multiple HR factors and that there is no difference in the extent of HR-mediated repair of the long template and short template HR reporters. Collectively, these data suggest that subtle changes in DSBR pathway proficiency may be observed when comparing the impact of both knockout and knockdown strategies e.g. through cell line adaptation or in different cell line backgrounds.

We also investigated whether the nature of the DSB ends affects the repair efficiency of the reporter substrate. The DSB can be generated by I-SceI or HindIII giving rise to non-cohesive 3′ overhangs or cohesive 5′ overhangs, respectively (Figure S3A) (29,33). Upon transfection of these reporters into DLD-1 BRCA2 KO cells, the repair of either substrate was similarly reduced suggesting that the DSB polarity and complementarity of the DSB ends do not affect HR-mediated repair efficiency in these assays (Figure S3B) and rules out a significant impact of the ligation of cohesive versus non-cohesive ends. However, modifying DSB end character in this way could be useful to understand differences in end-processing factors. Defective HR could also be observed in a format of the assay wherein the intact plasmid encoding the reporter substrate was co-transfected with a separate plasmid expressing I-SceI (Figure S3B). This opens the possibility of using these reporter substrates in contexts where intracellular generation of the DSB is required as has been previously reported for other DSBR reporter systems (34).

### Generation and validation of an SSA reporter assay substrate

**Figure 4.**
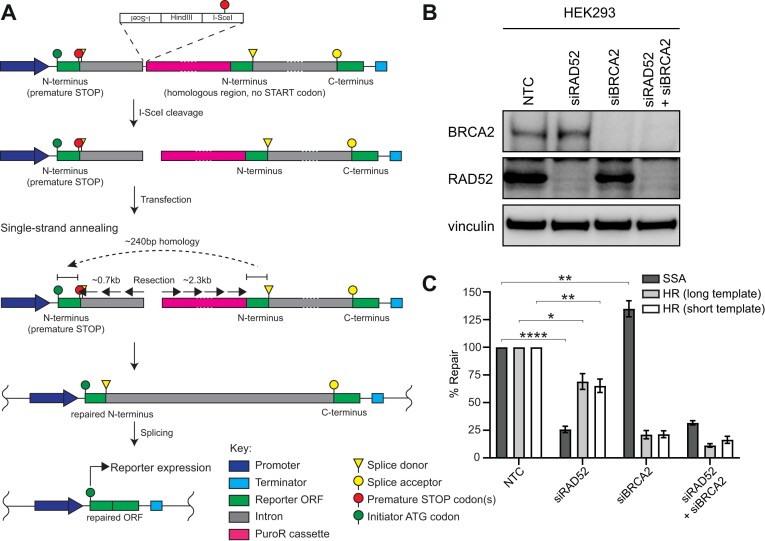
Schematic of extrachromosomal SSA reporter assay substrate and its genetic validation. (**A**) Schematic representation of the SSA reporter substrate. I-SceI cleavage generates a non-cohesive DSB in an intronic region downstream of the N-terminal portion of the nanoluciferase which harbours a premature stop codon. Upon transfection, SSA-mediated repair of the break involves bidirectional long-range resection to reveal ∼240 bp regions of homology that restore an intact N-terminal fragment and delete the intervening sequence. Upon repair, the N- and C-terminal portions of the reporter gene are spliced together reconstituting an ORF expressing NanoLuc. (**B**) Western blot confirming siRNA-mediated knockdown of RAD52 and BRCA2, alone or in combination in HEK293 cells. NTC denotes non-targeting control. (**C**) After RAD52 knockdown, cells are unable to perform SSA-mediated repair of the SSA reporter shown in (A) but are proficient in HR-mediated repair of both the HR reporters shown in Figure 2. NanoLuc luminescence was normalised to Firefly luminescence to determine substrate repair 24 h post-transfection. % repair of siRNA-treated cells is expressed relative to cells treated with a non-targeting control siRNA. Data represent mean ± SEM of three biological replicates, each averaging 8 technical replicates. Significance was determined by a Student's *t*-test (two-tailed, unpaired).

We also generated an extrachromosomal SSA reporter with the layout shown in Figure 4A. The engineered DSB is generated by I-SceI cleavage, which produces non-cohesive ends. The N-terminal portion of nanoluciferase is located proximal to the promoter in an exon with a premature stop codon and the homologous template for its repair is in a downstream exon and lacks a functional start codon. Successful SSA-mediated repair requires substantial resection either side of the break to reveal these exons in a ssDNA context suitable to align the extended ∼240 bp homology across the two N-terminal portions. This generates an intact N-terminal exon which can be spliced to the C-terminal portion to generate a functional nanoluciferase ORF.

To validate the dependency of the reporter on SSA, we tested the impact of knockdown of RAD52, a key component of SSA machinery (12,28) and BRCA2. The reporter substrate was transfected into HEK293 cells in which either RAD52, BRCA2 or both proteins were depleted by siRNA (Figure 4B). As expected, loss of RAD52 significantly reduced repair of the reporter by ∼75% **(**Figure 4C) (26). A mild increase in repair was observed upon loss of BRCA2 in agreement with previous reports (35). Co-depletion of RAD52 and BRCA2 did not further impact SSA-mediated repair (35). We observed a reciprocal impact in our HR reporter assay where knockdown of BRCA2 decreased the signal by ∼75% while the impact of RAD52 was much less pronounced with a signal decrease of ∼25% (Figure 4C) (36). Their roles as mediators of independent HDR pathways underpins the observed synthetic lethal relationship between RAD52 and BRCA2 (36,37).

### Pharmacological sensitivity of NHEJ and HR reporters

Having validated the function and repair pathway specificity of these extrachromosomal reporter assays, we next evaluated if the signals were both titratable and sensitive to small molecule inhibition using established tool compounds. Firstly, we evaluated the modulation of DSBR pathways by AZD7648, a potent and selective small molecule inhibitor of DNA-PKcs (38).

**Figure 5.**
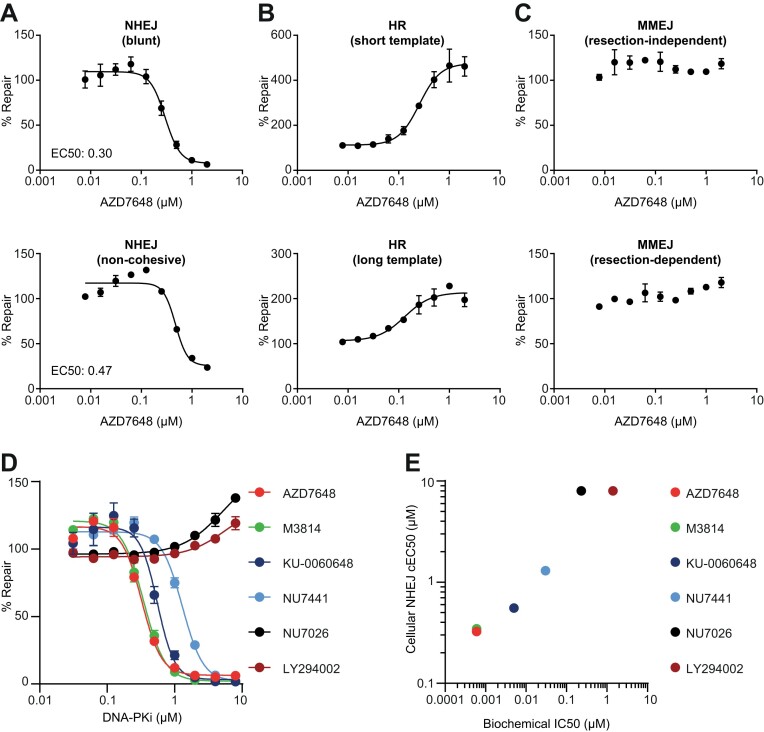
Pharmacological inhibition of DSB repair by DNA-PKcsi AZD7648. (A–C) HEK293 cells were transfected with the indicated extrachromosomal substrates and a Firefly luciferase plasmid (transfection control) and treated with increasing concentrations of DNA-PKcs inhibitor AZD7648. NanoLuc luminescence was normalised to Firefly luminescence to determine substrate repair 16 h post-transfection. Percentage inhibition was calculated relative to the DMSO control. Inhibition of DNA-PKcs caused a dose-dependent suppression of NHEJ (**A**) and a dose dependent up-regulation of HR (**B**), but did not robustly affect MMEJ (**C**). Data represent mean ± SEM of two biological replicates, each comprising eight technical replicates. NanoLuc and Firefly luminescence signals are shown in Figure S4A. (**D**) Indicated DNA-PKcs inhibitor compounds were tested in the blunt NHEJ reporter assay as described in (A). AZD7648, M3814, KU-0060648 and NU7441 inhibited NHEJ with EC50s of 0.33, 0.35, 0.56 and 1.3 μM, respectively. NU7026 and LY294002 did not inhibit NHEJ. Data represent mean ± SEM of two biological replicates, each comprising four technical replicates. NanoLuc and Firefly luminescence signals are shown in Figure S4B. (**E**) Correlation of cellular EC50 determined from the NHEJ reporter assay with previously reported biochemical IC50 values from DNA-PKcs autophosphorylation assays. EC50 values for NU7026 and LY294002 are plotted as the maximum concentration tested.

AZD7648 showed a clear, titratable inhibition of NHEJ in both the blunt and non-cohesive end variant of the non-blunt end NHEJ reporter assays in HEK293 cells (Figure 5A), and a clear stimulation of HR in both HR reporter assays (Figure 5B). No significant modulation of MMEJ was observed (Figure 5C). The observed patterns of DSBR modulation were driven specifically by changes in the NanoLuc signal generated by repair of the reporter rather than any effect of the inhibitor on transfection or viability as the Firefly control signal was relatively unperturbed (Figure S4A). Furthermore, the EC_50_s derived from the NHEJ assays (0.298 μM and 0.472 μM in the blunt and non-cohesive NHEJ reporters, respectively) are within 3–5-fold of the reported IC_50_ of suppression of DNA-PK autophosphorylation in cells (38) and provides an indication of cellular potency in a functional NHEJ assay.

We also used the NHEJ reporter assays as a method to confirm AZD7648 on-target inhibition by using an isogenic pair of HCT116 cell lines in which *PRKDC*, the gene encoding DNA-PKcs, was deleted, as confirmed by WB (Figure S5A). Consistent with the data obtained in HEK293 (Figure 5A), AZD7648 titratably inhibited NHEJ as assessed using both the blunt and non-cohesive end reporters, in wild-type cells (Figure S5B and C). However, no inhibitory effect was observed in PRKDC^−/−^ cells, which already had a baseline NHEJ reporter signal reduced by more than 90% in both the assays (Figure S5B–E). Furthermore, AZD7648 was observed to reduce NHEJ in wild-type cells to levels indistinguishable from PRKDC^−/−^ cells. A more detailed analysis of NanoLuc and Firefly levels showed that NanoLuc levels were not impacted in PRKDC^−/−^ cells in the absence of the target, supporting the high specificity of AZD7648-mediated inhibition of NHEJ through DNA-PKcs (Figure S5F and G).

We further expanded on these observations by testing a range of DNA-PKcs inhibitor molecules to understand if we could differentiate their cellular potency using the blunt NHEJ reporter assay. Four of the six compounds tested (AZD7648, M3814, KU-0060648 and NU7441) showed clear dose-dependent inhibition of NHEJ (Figures 5D, S4B). Two compounds (NU7026 and the broad spectrum PI3K inhibitor LY294002) showed no inhibition at the concentrations tested. Importantly, the cellular potency of the tested inhibitors in this cell-based assay correlated well with previously reported values for their inhibition in biochemical assays (Figure 5E) (38–41) and support the potential early application of these assays in cellular cascades for small molecule drug discovery.

To assess pharmacological sensitivity of the HR reporter assay, we used CAM833, a recently identified small molecule inhibitor that disrupts the BRCA2-RAD51 interaction and thus impairs HR (42). CAM833 inhibited the HR reporter assay in a dose-dependent manner (Figure S6A and B) with an EC_50_ of 3.3 μM. This inhibition of the HR reporter assay was also confirmed by a titratable inhibition of IR-induced RAD51 foci (Figure S6C and D), an orthogonal assessment of HR proficiency. Both values are in close agreement with the previously reported EC_50_ of inhibition of IR-induced RAD51 foci (42). Upon evaluation of the NanoLuc and Firefly levels, in addition to a clear dose-responsive inhibition of the NanoLuc reporter signal, we observed a reduction of the Firefly signal, possibly indicating cellular toxicity which could be an on-target consequence of HR inhibition. Therefore, these assays may yield additional information about small molecule effects, as well as serving as a functional assay, and further highlights the need to evaluate the effects of both the NanoLuc and Firefly components of the reporter signals.

Collectively these data suggest that these assays are sensitive to small molecule inhibition, are titratable and can be used to confirm both on-target and on-mechanism specificity. Critically for small molecule drug discovery, they can generate cellular EC_50_ values that can be used to rank compounds based on their cellular potency.

### Pharmacological sensitivity of MMEJ reporter assay

**Figure 6.**
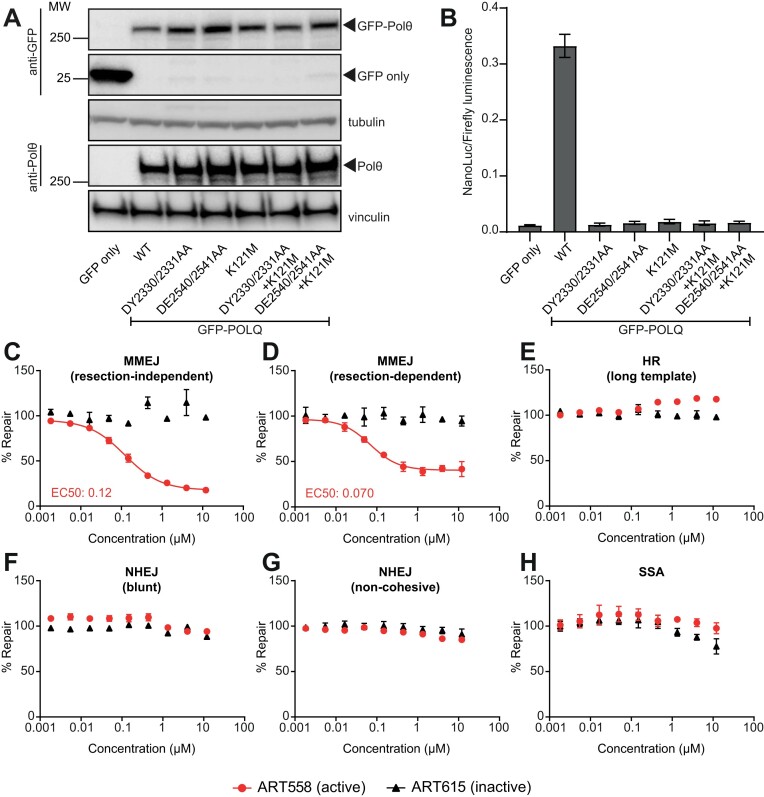
An inhibitor of Polθ polymerase specifically inhibits cellular MMEJ. (**A**) Western blot confirming transient overexpression of GFP-Polθ variants in U-2 OS POLQ^−/−^ cells. Indicated variants are defective in polymerase activity (DY2330/2331AA or DE2540/2541AA) or helicase activity (K121M), or both, as indicated. Tubulin and vinculin serve as loading controls for anti-GFP and anti-Polθ blots, respectively. (**B**) The elimination of MMEJ in U-2 OS POLQ^−/−^ cells is rescued by overexpression of wild-type GFP-Polθ but not by mutants defective in the polymerase or helicase domains, either alone or in combination. Data represent mean ± SEM of two biological replicates each averaging eight technical repeats. (C–H) HEK293 cells were transfected with the indicated extrachromosomal substrates and a Firefly luciferase plasmid (transfection control) and treated with increasing concentrations of an active inhibitor of Polθ (ART558) or an inactive control (ART615). NanoLuc luminescence was normalised to Firefly luminescence to determine substrate repair 24 h post-transfection. Percentage inhibition was calculated relative to the DMSO control. ART558 caused a dose-dependent inhibition of both resection-independent (**C**) and -dependent (**D**) MMEJ but not NHEJ (**E, F**), HR (**G**) or SSA (**H**). ART615 did not have any effect on DSB repair. Data represent mean ± SEM of two biological replicates, each averaging four technical replicates.

Having initially demonstrated that the MMEJ reporter assays are sensitive to genetic ablation of POLQ, we next sought to confirm that it would be specifically sensitive to inactivation of the catalytic activities of Polθ. U-2 OS POLQ^−/−^ cells were transfected with constructs expressing full length Polθ, either wild type or with point mutations that are known to inactivate the polymerase or helicase-like ATPase domains, alone or in combination (10,43,44). Transient expression of these constructs at similar levels was confirmed by Western blot (Figure 6A). We then used the resection-independent MMEJ reporter assay to understand the impact of these mutations on MMEJ-mediated repair. Strikingly, only wild type Polθ was able to restore the MMEJ repair capacity of the POLQ KO cells (Figure 6B). Polymerase and/or helicase-dead mutants were not able to rescue the defect, indicating that both activities are essential for the repair of this substrate. As the assay is sensitive to inactivation of the polymerase domain, these data suggested that it would be suitable to screen for small molecule inhibitors of the polymerase domain of Polθ (20).

We tested Polθ inhibitor ART558 and an inactive enantiomer, ART615 (21), across the full panel of DSBR reporters to demonstrate that they specifically inhibited MMEJ. ART558 robustly inhibited both MMEJ reporter assays in a dose-responsive manner with an EC_50_ of 0.12 μM and 0.070 μM in the resection-independent and resection-dependent reporter, respectively (Figure 6C and D). No MMEJ inhibition was observed with ART615. The extent of maximum inhibition of the resection-dependent reporter was not complete, in agreement with the genetic dependency of this reporter on Polθ (Figure 1K). No inhibitory effects were observed on HR, NHEJ or SSA (Figure 6E–H), suggesting that inhibition of Polθ polymerase domain by ART558 specifically inhibits MMEJ and that the inhibitor had no off-target effects on alternative DSBR pathways.

We also sought to evaluate the potential of the MMEJ assays to report on small molecule inhibitors of the Polθ helicase-like ATPase domain. A recent report has proposed novobiocin as a first-in-class inhibitor of Polθ targeting the helicase activity (45). We tested novobiocin alongside two additional Polθ helicase inhibitors, POLθ-HDi1 and POLθ-HDi2 (disclosed in patent WO 2020/243459 and used in (46)). We observed that novobiocin equally affected both the control Firefly and reporter NanoLuc signals, which may indicate possible cellular toxicity (Figure S7, (45)) that cannot be distinguished from specific inhibition of repair. However, both POLθ-HDi1 and POLθ-HDi2 were able to specifically inhibit MMEJ with similar potency to ART558, showing clear separation in their ability to affect the reporter NanoLuc and control Firefly signals, and confirming that pharmacological targeting of the helicase domain is sufficient to inhibit MMEJ in cells (Figure S7). Intriguingly, the MMEJ EC_50_ Hill slope for both POLθ-HDi1 and POLθ-HDi2 is < 1, indicating negative cooperativity.

The MMEJ reporter assays were thus demonstrated to be a sensitive and highly selective cellular assay, dependent on Polθ polymerase and helicase enzymatic activities. Furthermore, the assays can be used in a plate-based format to provide information on cellular potency and specificity of small molecule inhibitors of Polθ for guiding drug discovery.

## Discussion

Efforts to identify and validate small molecules targeting DSBR significantly benefit from robust, reproducible, quantitative and titratable mechanistic cellular assays suitable for the determination of cellular potency and ranking of compounds. We report here a validated suite of extrachromosomal DSBR reporter assays suitable for the assessment of MMEJ, cNHEJ, HR and SSA integrity in cells. These assays can be used in multiple contexts including but not limited to discovery and validation of small molecules targeting DSBR factors.

Through a genetic approach, we have demonstrated that all these assays specifically depend on key components of the pathways they were designed to measure (Figures 1–4). In addition, we have shown that the NHEJ, HR and MMEJ assays are sensitive to pharmacological modulation and titration (Figures 5A, 6C, D and S6A) allowing the generation of dose-response curves to quantify cellular potency of small molecules. Assessment of small molecules across a panel of assays can be used to demonstrate DSBR pathway specificity (Figures 5A–C and 6C–H), providing evidence of on-pathway mechanism, and application in KO models provides evidence of on-target mechanism (Figure S5).

Pharmacological validation of the HR and NHEJ assays with existing small molecule inhibitors of BRCA2-RAD51 and DNA-PKcs demonstrated that the measured potency in these reporter assays aligned well with orthogonal assays such as the suppression of RAD51 foci or DNA-PKcs autophosphorylation, respectively (Figures S6 and 5E). The potency of a range of DNA-PKcs inhibitors in an NHEJ reporter assay additionally correlated with their biochemical IC_50_ (Figure 5D and E), supporting the applicability of these assays to correctly rank compounds in a cellular assay and exemplifies the potential to use these assays as part of cellular screening cascades. Indeed, in our previous work, we have utilised the resection-independent MMEJ assay (Figure 1A) to identify and develop highly potent and specific small molecule inhibitors of the polymerase domain of Polθ (20). The assay is genetically dependent on the presence of Polθ as well as its two main enzymatic functions, a polymerase and a helicase, and thus explicitly reports on the activity of Polθ performing its native cellular repair function (Figures 1A, F, G, 6A, B). The assay allowed us to rapidly identify cell permeable small molecules that specifically inhibit Polθ-mediated MMEJ whilst leaving other DSBR pathways unperturbed, demonstrating pathway selectivity (Figure 6C–H). Generation of robust cellular EC_50_s allowed compound ranking to both understand potency differences between biochemical and phenotypic assays and help drive structure-activity relationship (SAR) (20). Further mechanistic and phenotypic analyses have demonstrated that these Polθ inhibitors directly modulate Polθ in cells and selectively kill HRD cells, phenocopying genetic loss of POLQ. Critically, these compounds also enhance PARPi-mediated cell killing, target PARPi resistant cell lines and tumours resulting from loss of 53BP1 and Shieldin components and have been recently shown to potentiate radiotherapy (21,47).

We were unable to demonstrate conclusively that novobiocin specifically inhibits MMEJ in cells, but this was not the case for ART558 or two helicase inhibitors, POLθ-HDi1 and POLθ-HDi2 (Figure S7). However, the data obtained in these extrachromosomal MMEJ assays do not rule out the possibility that novobiocin inhibits cellular MMEJ. Novobiocin treatment causes a clear reduction in the reporter NanoLuc signal. However, as the compound equally affects the control Firefly luciferase signal, we cannot conclude that the effect is specific and possibly highlights its potentially promiscuous pharmacology (46). Modulation of Polθ-mediated MMEJ by novobiocin may however reflect a more complicated mechanism of action not captured in the extrachromosomal assay format (48) and/or one that is revealed in a different context such as a chromosomally integrated MMEJ reporter system (45).

The reporter assays described in this study use extrachromosomal linear substrates that are complementary to chromosomal reporter systems. There are a number of pros and cons to using these assays as described below and summarised in Supplementary Table 7.

There are various advantages to using NanoLuc based extrachromosomal assays. Firstly, these linearised substrates can be transfected into cells using standard methods and are not limited to a specific cell line providing broad applicability for various uses. For example, we have used them successfully in a range of isogenic models in different backgrounds (Figures 1–4), but also show consistent quantifiable impact in genetic studies (MMEJD status in eHAP1 and U-2 OS, Figure 1F, J) and pharmacological studies (DNA-PKcs inhibition in HEK293 and HCT116, Figures 5A, S5B, S5C). Although optimisation of transfection is a prerequisite to ensure robust introduction of the extrachromosomal substrate into cells, both lipofection and nucleofection strategies can be used (Figure S8) and the latter may expand the use of these assays in traditionally hard to transfect cell lines such as suspension and primary cells.

Secondly, with the exception of the resection-independent MMEJ reporter, the DSB is enzymatically introduced by a single restriction digest reaction prior to transfection and hence the DSB is instantly available for cellular repair. The pre-transfection restriction enzyme digest reaction is simple and highly efficient. Repair signals are only detected upon transfection with pre-digested reporter substrates, and any residual uncut plasmid does not generate significant background NanoLuc signals (Figure S9). Chromosomal reporter assays are reliant on intracellular endonucleolytic cleavage upon Cas9 or I-SceI induction. This approach can be optimised for efficient cutting to generate DSBs, however, it limits studies to specific cell lines engineered to express these endonucleases and gRNA features, chromatin status and cell line choice can affect nuclease cutting efficiency (49).

Thirdly, extrachromosomal reporter assays can be run and read out in under 24 h thus capturing specific and rapid repair responses with sufficient sensitivity. The simple and fast turnaround of these assays makes them amenable to high throughput screening in a drug discovery setting and less prone to artefacts that can arise from general toxicity and cell cycle perturbations induced by the effects of long-term exposure to small molecules or gene depletion. Chromosomally integrated, fluorescence-based DSBR reporters are usually reliant on a 48–96 h assay window. Although this may not always be an issue, a complementary orthogonal reporter system that requires a shorter incubation time may be useful in contexts where timepoints are restrictive owing to adverse or unexpected cell perturbations or to increase assay turnaround and throughput. Supporting this, the extrachromosomal resection-independent MMEJ assay can generate robust and reliable EC_50_s in as little as 6 h (Figure S10).

Fourthly, the use of a luminescence-based reporter system makes these assays amenable to a medium-high throughput format that can be run using a plate-reader. The assays described all use the NanoLuc and Firefly luminescence, which allows ease of set-up for multiple DSBR assays, with a consistent format. In situ luminescence detection also improves upon the sample handling requirements for flow cytometric analyses of fluorescent systems. Performing higher throughput assays is also facilitated by straightforward substrate production as the parental plasmids can be propagated easily in bacteria, making reporter substrate generation easily scalable for increased assay throughput. Whilst the ability to perform high throughput DSBR assays is a significant advantage of the reporters described in this study, this has also been achieved using chromosomal reporter assays in both imaging and flow-cytometry-based screens, although this approach is again limited to an engineered cell line incorporating the reporter and requires suitable equipment (50,51).

Finally, the reporters can be functionalised to report on mechanistic subtleties of DSBR. For examples, the DSB ends in non-blunt NHEJ (Figure 2B) and long-template HR (Figure 3A) reporters can be cohesive or non-cohesive dependent on restriction enzyme used. Also, the resection-independent reporter structurally mimics pre-resected termini with perfect terminal microhomologies unlikely to need additional processing prior to Polθ-mediated repair (9,10) and is almost completely abolished by loss of Polθ or even inactivating mutations in the polymerase and/or helicase domain. Intriguingly, we observed a residual MMEJ signal (∼40%) in the resection-dependent MMEJ reporter upon genetic ablation or pharmacological inhibition of Polθ (Figures 1K and 6D). This suggests that other as yet unknown factors may prosecute MMEJ in the absence of Polθ, on substrates where an alternative pathway choice may be triggered by a context primed at an upstream stage in the repair pathway.

The resection-independent MMEJ reporter assay likely represents an exquisitely Polθ-specific structural repair context capturing a post-resection stage in MMEJ that cannot stably be generated by a chromosomal reporter system, making this reporter substrate uniquely deployable in an extrachromosomal format. This strategy also exemplifies how these extrachromosomal reporters could be further modified to generate other unique target-dependent structures or repair-ready intermediates and lesions to identify pathway components or small molecule inhibitors of these pathways. Approaches to introduce functionalised DSBs in chromosomal reporters using Cas9 variants and modified guide spacing have been reported (52), but these are limited to specific cell lines expressing Cas9. More complex functionalisation, such as that achieved in the resection-independent reporter, may ultimately be more tractable using extrachromosomal reporter substrates.

The reporters described in this study have a wide range of applications because of their robust and sensitive luminescence-based readout and utility in a range of cell models limited only by their transfectability.

Although advantages, adaptations and applications of the extrachromosomal luminescence-based DSBR reporters have been described, they have additional limitations compared to existing chromosomal reporter systems.

The consistent incorporation of NanoLuc and Firefly luminescence supports the implementation of a range of these assays but it does limit their application to their use in isolation. A central feature of DSBR is the complexity of the repair pathway selection, redundancy and compensation. Whereas we have demonstrated that the reporters can read out such responses, they do not do so simultaneously and highlights the value of multi-pathway chromosomal reporters such as the Traffic Light Reporter (51) and DSB-Spectrum systems (53).

Additionally, our genetic and pharmacological validation confirms sensitivity of each reporter to loss or inhibition of core machinery of their cognate repair pathways, however it remains unclear how fully they recapitulate physiological contexts of DSBR such as regulatory signalling, peripheral repair machinery and chromatinisation. Chromosomal reporter systems comprise an engineered DSB in a chromatin context and are thus more likely to replicate a repair response in its native context.

Furthermore, whilst the ability to introduce extrachromosomal reporter substrates into cells through transient transfection is a major advantage, increasing their breadth of application and turnaround time, interpretation of repair of extrachromosomal reporters needs some caution as they may be non-physiological. Compared to chromosomal reporters, extrachromosomal reporters may be present at high levels (compared to single copy genomic integration), accessing repair machinery outside of cell cycle regulation, or upsetting the balance of repair factors through their sequestration. The range of chromosomal reporters is also constantly expanding (reviewed in (18)) and captures mechanistic details and sub-pathways of DSBR that have not yet been recapitulated with extrachromosomal systems. Thus, we anticipate that these extrachromosomal reporters will orthogonally expand the toolkit of available assays beyond existing DSBR reporter systems, which have been largely dominated by chromosomally integrated reporters.

In summary, we have presented the versatile applications of a suite of extrachromosomal DSBR reporter assays sensitive to genetic and pharmacological modulation. These include interrogating genetic impact on DSBR proficiency, and the identification and validation of small molecules. The latter strategies provide insight into on-target/on-mechanism activity, specificity, and cellular potency, to inform compound ranking, which are critical for cellular screening cascades to identify DDR modulators. Future studies will explore the range of applications in which these reporter assays can be used (such as target identification or screening for small molecules against other DSBR targets), more complex readouts (sequencing of recovered substrates post-repair to assess InDel incorporation) and additional modifications that further functionalise the substrates to understand DSBR for both basic research and drug discovery.

## Supplementary Material

gkad1196_Supplemental_File

## Data Availability

All data needed to evaluate the conclusions of this study are presented in the article and/or the Supplementary Data. The data will be shared on reasonable request to the corresponding author.

## Notes

Present address: Graeme Hewitt, School of Cancer & Pharmaceutical Sciences, King's College London, London SE1 9RT, UK.

## References

[1] Jackson S.P., Bartek J. The DNA-damage response in human biology and disease. Nature. 2009; 461:1071–1078. 19847258 10.1038/nature08467 PMC2906700

[2] Scully R., Panday A., Elango R., Willis N.A. DNA double-strand break repair-pathway choice in somatic mammalian cells. Nat. Rev. Mol. Cell Biol. 2019; 20:698–714. 31263220 10.1038/s41580-019-0152-0 PMC7315405

[3] Verma P., Greenberg R.A. Noncanonical views of homology-directed DNA repair. Genes Dev. 2016; 30:1138–1154. 27222516 10.1101/gad.280545.116 PMC4888836

[4] Ramsden D.A., Carvajal-Garcia J., Gupta G.P. Mechanism, cellular functions and cancer roles of polymerase-theta-mediated DNA end joining. Nat. Rev. Mol. Cell Biol. 2022; 23:125–140. 34522048 10.1038/s41580-021-00405-2 PMC13537726

[5] Black S.J., Kashkina E., Kent T., Pomerantz R.T. DNA polymerase θ: a unique multifunctional end-joining machine. Genes (Basel). 2016; 7:67. 27657134 10.3390/genes7090067 PMC5042397

[6] Zahn K.E., Jensen R.B. Polymerase θ coordinates multiple intrinsic enzymatic activities during DNA repair. Genes (Basel). 2021; 12:1310. 34573292 10.3390/genes12091310 PMC8470613

[7] Wood R.D., Doublié S. Genome protection by DNA polymerase θ. Annu. Rev. Genet. 2022; 56:207–228. 36028228 10.1146/annurev-genet-072920-041046 PMC10351424

[8] Kent T., Chandramouly G., Mcdevitt S.M., Ozdemir A.Y., Pomerantz R.T. Mechanism of microhomology-mediated end-joining promoted by human DNA polymerase θ. Nat. Struct. Mol. Biol. 2015; 22:230–237. 25643323 10.1038/nsmb.2961 PMC4351179

[9] Black S.J., Ozdemir A.Y., Kashkina E., Kent T., Rusanov T., Ristic D., Shin Y., Suma A., Hoang T., Chandramouly G. et al. Molecular basis of microhomology-mediated end-joining by purified full-length Polθ. Nat. Commun. 2019; 10:4423. 31562312 10.1038/s41467-019-12272-9 PMC6764996

[10] Wyatt D.W., Feng W., Conlin M.P., Yousefzadeh M.J., Roberts S.A., Mieczkowski P., Wood R.D., Gupta G.P., Ramsden D.A. Essential roles for polymerase θ-mediated end joining in the repair of chromosome breaks. Mol. Cell. 2016; 63:662–673. 27453047 10.1016/j.molcel.2016.06.020 PMC4992412

[11] Mateos-Gomez P.A., Kent T., Deng S.K., McDevitt S., Kashkina E., Hoang T.M., Pomerantz R.T., Sfeir A. The helicase domain of Polθ counteracts RPA to promote alt-NHEJ. Nat. Struct. Mol. Biol. 2017; 24:1116–1123. 29058711 10.1038/nsmb.3494 PMC6047744

[12] Bhargava R., Onyango D.O., Stark J.M. Regulation of single-strand annealing and its role in genome maintenance. Trends Genet. 2016; 32:566–575. 27450436 10.1016/j.tig.2016.06.007 PMC4992407

[13] Anand R., Buechelmaier E., Belan O., Newton M., Vancevska A., Kaczmarczyk A., Takaki T., Rueda D.S., Powell S.N., Boulton S.J. HELQ is a dual-function DSB repair enzyme modulated by RPA and RAD51. Nature. 2022; 601:268–273. 34937945 10.1038/s41586-021-04261-0 PMC8755542

[14] Zhao B., Rothenberg E., Ramsden D.A., Lieber M.R. The molecular basis and disease relevance of non-homologous DNA end joining. Nat. Rev. Mol. Cell Biol. 2020; 21:765–781. 33077885 10.1038/s41580-020-00297-8 PMC8063501

[15] O’Connor M.J. Targeting the DNA damage response in cancer. Mol. Cell. 2015; 60:547–560. 26590714 10.1016/j.molcel.2015.10.040

[16] Curtin N.J. Targeting the DNA damage response for cancer therapy. Biochem. Soc. Trans. 2023; 51:207–221. 36606678 10.1042/BST20220681 PMC9988002

[17] Groelly F.J., Fawkes M., Dagg R.A., Blackford A.N., Tarsounas M. Targeting DNA damage response pathways in cancer. Nat. Rev. Cancer. 2023; 23:78–94. 36471053 10.1038/s41568-022-00535-5

[18] van de Kooij B., van Attikum H. Genomic reporter constructs to monitor pathway-specific repair of DNA double-strand breaks. Front. Genet. 2021; 12:809832. 35237296 10.3389/fgene.2021.809832 PMC8884240

[19] Gunn A., Stark J.M. I-SceI-based assays to examine distinct repair outcomes of mammalian chromosomal double strand breaks. Methods Mol. Biol. 2012; 920:379–391. 22941618 10.1007/978-1-61779-998-3_27

[20] Stockley M.L., Ferdinand A., Benedetti G., Blencowe P., Boyd S.M., Calder M., Charles M.D., Edwardes L.V., Ekwuru T., Finch H. et al. Discovery, characterization, and structure-based optimization of small-molecule in vitro and in vivo probes for human DNA polymerase theta. J. Med. Chem. 2022; 65:13879–13891. 36200480 10.1021/acs.jmedchem.2c01142

[21] Zatreanu D., Robinson H.M.R., Alkhatib O., Boursier M., Finch H., Geo L., Grande D., Grinkevich V., Heald R.A., Langdon S. et al. Polθ inhibitors elicit BRCA-gene synthetic lethality and target PARP inhibitor resistance. Nat. Commun. 2021; 12:3636. 34140467 10.1038/s41467-021-23463-8 PMC8211653

[22] Belan O., Sebald M., Adamowicz M., Anand R., Vancevska A., Neves J., Grinkevich V., Hewitt G., Segura-Bayona S., Bellelli R. et al. POLQ seals post-replicative ssDNA gaps to maintain genome stability in BRCA-deficient cancer cells. Mol. Cell. 2022; 82:4664–4680. 36455556 10.1016/j.molcel.2022.11.008

[23] Drzewiecka M., Barszczewska-Pietraszek G., Czarny P., Skorski T., Śliwiński T. Synthetic lethality targeting Polθ. Genes (Basel). 2022; 13:1101. 35741863 10.3390/genes13061101 PMC9223150

[24] Ceccaldi R., Liu J.C., Amunugama R., Hajdu I., Primack B., Petalcorin M.I.R., O’Connor K.W., Konstantinopoulos P.A., Elledge S.J., Boulton S.J. et al. Homologous-recombination-deficient tumours are dependent on Polθ-mediated repair. Nature. 2015; 518:258–262. 25642963 10.1038/nature14184 PMC4415602

[25] Hall M.P., Unch J., Binkowski B.F., Valley M.P., Butler B.L., Wood M.G., Otto P., Zimmerman K., Vidugiris G., Machleidt T. et al. Engineered luciferase reporter from a deep sea shrimp utilizing a novel imidazopyrazinone substrate. ACS Chem. Biol. 2012; 7:1848–1857. 22894855 10.1021/cb3002478 PMC3501149

[26] Bennardo N., Cheng A., Huang N., Stark J.M. Alternative-NHEJ is a mechanistically distinct pathway of mammalian chromosome break repair. PLoS Genet. 2008; 4:e1000110. 18584027 10.1371/journal.pgen.1000110 PMC2430616

[27] So A., Dardillac E., Muhammad A., Chailleux C., Sesma-Sanz L., Ragu S., Le Cam E., Canitrot Y., Masson J.Y., Dupaigne P. et al. RAD51 protects against nonconservative DNA double-strand break repair through a nonenzymatic function. Nucleic Acids Res. 2022; 50:2651–2666. 35137208 10.1093/nar/gkac073 PMC8934640

[28] Stark J.M., Pierce A.J., Oh J., Pastink A., Jasin M. Genetic steps of mammalian homologous repair with distinct mutagenic consequences. Mol. Cell. Biol. 2004; 24:9305–9316. 15485900 10.1128/MCB.24.21.9305-9316.2004 PMC522275

[29] Mao Z., Bozzella M., Seluanov A., Gorbunova V. Comparison of nonhomologous end joining and homologous recombination in human cells. DNA Repair (Amst.). 2008; 7:1765–1771. 18675941 10.1016/j.dnarep.2008.06.018 PMC2695993

[30] Pierce A.J., Johnson R.D., Thompson L.H., Jasin M. XRCC3 promotes homology-directed repair of DNA damage in mammalian cells. Genes Dev. 1999; 13:2633–2638. 10541549 10.1101/gad.13.20.2633 PMC317094

[31] Stark J.M., Pierce A.J., Oh J., Pastink A., Jasin M. Genetic steps of mammalian homologous repair with distinct mutagenic consequences. Mol. Cell. Biol. 2004; 24:9305–9316. 15485900 10.1128/MCB.24.21.9305-9316.2004 PMC522275

[32] Pierce A.J., Hu P., Han M., Ellis N., Jasin M. Ku DNA end-binding protein modulates homologous repair of double-strand breaks in mammalian cells. Genes Dev. 2001; 15:3237–3242. 11751629 10.1101/gad.946401 PMC312854

[33] Seluanov A., Mao Z., Gorbunova V. Analysis of DNA double-strand break (DSB) repair in mammalian cells. J. Vis. Exp. 2010; 43:2002. 10.3791/2002 PMC3157866 20864925

[34] Chen C., Avdievich E., Zhang Y., Zhang Y., Wei K., Lee K., Edelmann W., Jasin M., LaRocque J.R. EXO1 suppresses double-strand break induced homologous recombination between diverged sequences in mammalian cells. DNA Repair (Amst.). 2017; 57:98–106. 28711786 10.1016/j.dnarep.2017.07.003 PMC5584059

[35] Han J., Ruan C., Huen M.S.Y., Wang J., Xie A., Fu C., Liu T., Huang J. BRCA2 antagonizes classical and alternative nonhomologous end-joining to prevent gross genomic instability. Nat. Commun. 2017; 8:1470. 29133916 10.1038/s41467-017-01759-y PMC5684403

[36] Feng Z., Scott S.P., Bussen W., Sharma G.G., Guo G., Pandita T.K., Powell S.N. Rad52 inactivation is synthetically lethal with BRCA2 deficiency. Proc. Natl. Acad. Sci. USA. 2011; 108:686–691. 21148102 10.1073/pnas.1010959107 PMC3021033

[37] Lok B.H., Carley A.C., Tchang B., Powell S.N. RAD52 inactivation is synthetically lethal with deficiencies in BRCA1 and PALB2 in addition to BRCA2 through RAD51-mediated homologous recombination. Oncogene. 2013; 32:3552–3558. 22964643 10.1038/onc.2012.391 PMC5730454

[38] Fok J.H.L., Ramos-Montoya A., Vazquez-Chantada M., Wijnhoven P.W.G., Follia V., James N., Farrington P.M., Karmokar A., Willis S.E., Cairns J. et al. AZD7648 is a potent and selective DNA-PK inhibitor that enhances radiation, chemotherapy and olaparib activity. Nat. Commun. 2019; 10:5065. 31699977 10.1038/s41467-019-12836-9 PMC6838110

[39] Munck J.M., Batey M.A., Zhao Y., Jenkins H., Richardson C.J., Cano C., Tavecchio M., Barbeau J., Bardos J., Cornell L. et al. Chemosensitization of cancer cells by KU-0060648, a dual inhibitor of DNA-PK and PI-3K. Mol. Cancer Ther. 2012; 11:1789–1798. 22576130 10.1158/1535-7163.MCT-11-0535 PMC3428850

[40] Zenke F.T., Zimmermann A., Sirrenberg C., Dahmen H., Kirkin V., Pehl U., Grombacher T., Wilm C., Fuchss T., Amendt C. et al. Pharmacologic inhibitor of DNA-PK, M3814, potentiates radiotherapy and regresses human tumors in mouse models. Mol. Cancer Ther. 2020; 19:1091–1101. 32220971 10.1158/1535-7163.MCT-19-0734

[41] Veuger S.J., Curtin N.J., Richardson C.J., Smith G.C.M., Durkacz B.W. Radiosensitization and DNA repair inhibition by the combined use of novel inhibitors of DNA-dependent protein kinase and poly(ADP-ribose) polymerase-1. Cancer Res. 2003; 63:6008–6015. 14522929

[42] Scott D.E., Francis-Newton N.J., Marsh M.E., Coyne A.G., Fischer G., Moschetti T., Bayly A.R., Sharpe T.D., Haas K.T., Barber L. et al. A small-molecule inhibitor of the BRCA2-RAD51 interaction modulates RAD51 assembly and potentiates DNA damage-induced cell death. Cell Chem. Biol. 2021; 28:835–847. 33662256 10.1016/j.chembiol.2021.02.006 PMC8219027

[43] Yoon J.-H., McArthur M.J., Park J., Basu D., Wakamiya M., Prakash L., Prakash S. Error-prone replication through UV lesions by DNA polymerase θ protects against skin cancers. Cell. 2019; 176:1295–1309. 30773314 10.1016/j.cell.2019.01.023 PMC6453116

[44] Zahn K.E., Averill A.M., Aller P., Wood R.D., Doublié S. Human DNA polymerase θ grasps the primer terminus to mediate DNA repair. Nat. Struct. Mol. Biol. 2015; 22:304–311. 25775267 10.1038/nsmb.2993 PMC4385486

[45] Zhou J., Gelot C., Pantelidou C., Li A., Yücel H., Davis R.E., Färkkilä A., Kochupurakkal B., Syed A., Shapiro G.I. et al. A first-in-class polymerase theta inhibitor selectively targets homologous-recombination-deficient tumors. Nat. Cancer. 2021; 2:598–610. 34179826 10.1038/s43018-021-00203-x PMC8224818

[46] Wimberger S., Akrap N., Firth M., Brengdahl J., Engberg S., Schwinn M.K., Slater M.R., Lundin A., Hsieh P.-P., Li S. et al. Simultaneous inhibition of DNA-PK and Pol⊖ improves integration efficiency and precision of genome editing. Nat. Commun. 2023; 14:4761. 37580318 10.1038/s41467-023-40344-4 PMC10425386

[47] Rodriguez-Berriguete G., Ranzani M., Prevo R., Puliyadi R., Machado N., Bolland H.R., Millar V., Ebner D., Boursier M., Cerutti A. et al. Small-molecule Polθ inhibitors provide safe and effective tumor radiosensitization in preclinical models. Clin. Cancer Res. 2023; 29:1631–1642. 36689546 10.1158/1078-0432.CCR-22-2977 PMC10102842

[48] Syed A., Filandr F., Patterson-Fortin J., Bacolla A., Ravindranathan R., Zhou J., McDonald D.T., Albuhluli M.E., Verway-Cohen A., Newman J.A. et al. Novobiocin blocks nucleic acid binding to Polθ and inhibits stimulation of its ATPase activity. Nucleic Acids Res. 2023; 51:9920–9937. 37665033 10.1093/nar/gkad727 PMC10570058

[49] Konstantakos V., Nentidis A., Krithara A., Paliouras G. CRISPR-Cas9 gRNA efficiency prediction: an overview of predictive tools and the role of deep learning. Nucleic Acids Res. 2022; 50:3616–3637. 35349718 10.1093/nar/gkac192 PMC9023298

[50] Adamson B., Smogorzewska A., Sigoillot F.D., King R.W., Elledge S.J. A genome-wide homologous recombination screen identifies the RNA-binding protein RBMX as a component of the DNA-damage response. Nat. Cell Biol. 2012; 14:318–328. 22344029 10.1038/ncb2426 PMC3290715

[51] Certo M.T., Ryu B.Y., Annis J.E., Garibov M., Jarjour J., Rawlings D.J., Scharenberg A.M. Tracking genome engineering outcome at individual DNA breakpoints. Nat. Methods. 2011; 8:671–676. 21743461 10.1038/nmeth.1648 PMC3415300

[52] Bothmer A., Phadke T., Barrera L.A., Margulies C.M., Lee C.S., Buquicchio F., Moss S., Abdulkerim H.S., Selleck W., Jayaram H. et al. Characterization of the interplay between DNA repair and CRISPR/Cas9-induced DNA lesions at an endogenous locus. Nat. Commun. 2017; 8:13905. 28067217 10.1038/ncomms13905 PMC5227551

[53] van de Kooij B., Kruswick A., van Attikum H., Yaffe M.B. Multi-pathway DNA-repair reporters reveal competition between end-joining, single-strand annealing and homologous recombination at Cas9-induced DNA double-strand breaks. Nat. Commun. 2022; 13:5295. 36075911 10.1038/s41467-022-32743-w PMC9458747

