# Supplementary material for: Quantitative, titratable and high-throughput reporter assays to measure DNA double strand break repair activity in cells
Source: Nucleic Acids Res. 2023 Dec 18;52(4):1736–52. doi: 10.1093/nar/gkad1196 (PMC10899754; doi:10.1093/nar/gkad1196)
Supplement: gkad1196_Supplemental_File [file gkad1196_supplemental_file.pdf]

SUPPLEMENTARY DATA

Quantitative, titratable and high-throughput reporter assays to measure DNA double strand break repair activity in cells

SUPPLEMENTARY FIGURES

Figure S1. Genetic knockout of the Ligase IV complex is sufficient to eliminate repair of the blunt end NHEJ substrate

Figure S2. Gene silencing of BRCA2 in DLD-1 cells decreases repair of the HR reporter substrates

Figure S3. Repair of the HR reporter substrate is not impacted by method of DSB introduction

Figure S4. Pharmacological inhibition of DSB repair by DNA-PKcsi AZD7648 specifically modulates the NHEJ and HR reporter signals

Figure S5. Pharmacological inhibition of NHEJ reporter signal by DNA-PKcsi AZD7648 in WT and DNA-PKcs knockout cells

Figure S6. Pharmacological inhibition of HR by RAD51i CAM833

Figure S7. Evaluation of Polθ helicase inhibitors in MMEJ assays

Figure S8. Extrachromosomal substrates can be transfected into cells by lipofection or nucleofection

Figure S9. I-SceI pre-digested reporter substrates are required to generate NanoLuc signals upon transfection

Figure S10. Resection-independent MMEJ assays performed at multiple timepoints

SUPPLEMENTARY TABLES

Supplementary Table 1: Oligonucleotides
Supplementary Table 2: Cell lines
Supplementary Table 3: siRNA
Supplementary Table 4: Antibodies
Supplementary Table 5: Compounds
Supplementary Table 6: Software
Supplementary Table 7: Comparison of DSBR reporter systems

SUPPLEMENTARY REFERENCES

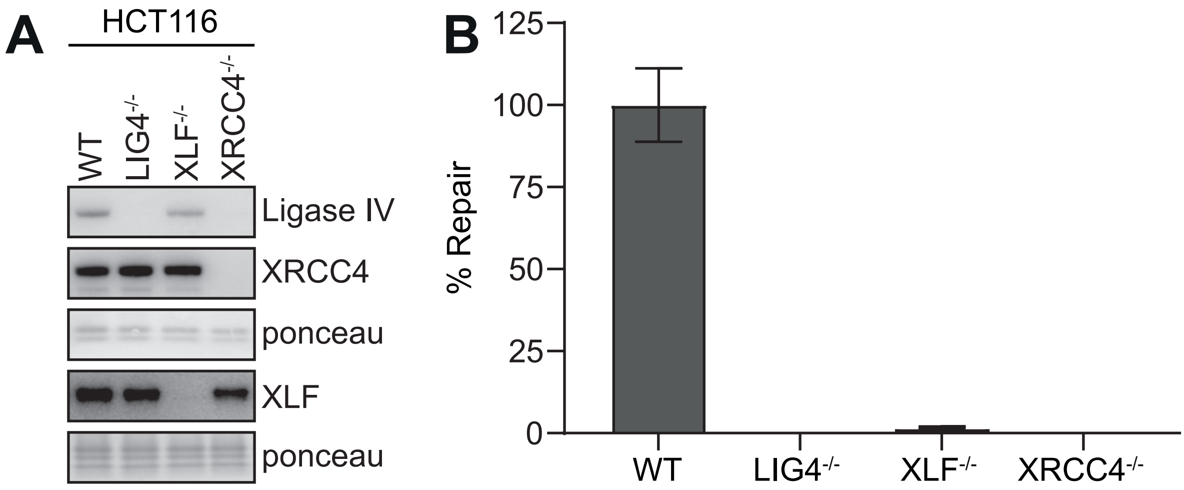

A
HCT116
WT
LIG4-/-
XLF-/-
XRCC4-/-
Ligase IV
XRCC4
ponceau
XLF
ponceau
B
125
100
75
50
25
0
% Repair
WT
LIG4-/-
XLF-/-
XRCC4-/-

Figure S1

Figure S1. Genetic knockout of the Ligase IV complex is sufficient to eliminate repair of the blunt end NHEJ substrate.

(A) Western blot of isogenic HCT116 cell lines confirming knockout of LIG4, XLF and XRCC4. $XRCC4^{-/-}$ cells show co-depletion of Ligase IV.

(B) Knockout of LIG4, XLF or XRCC4 ablates NHEJ-mediated repair of the substrate.

NanoLuc luminescence was normalised to Firefly luminescence (expressed from pGL4.53[luc2/PGK]) to determine substrate repair 24 h post-transfection. % repair of NHEJ knockout cells is expressed relative to the parental WT cell line. Data represent mean ± SD of 4 technical replicates.

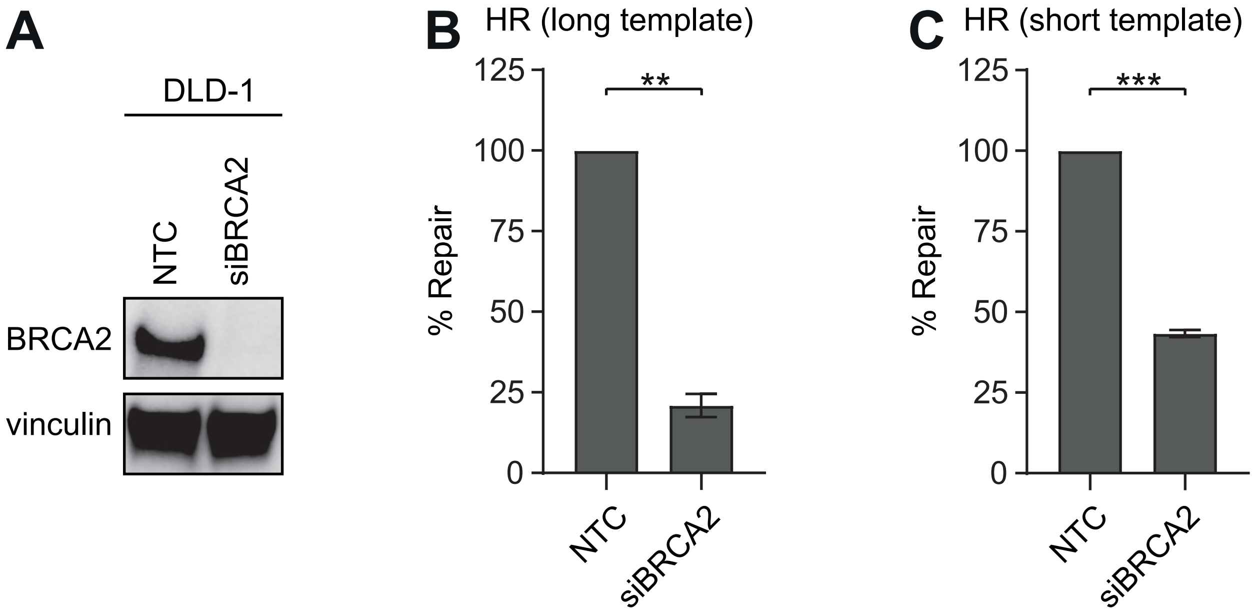
A
DLD-1
NTC
siBRCA2
BRCA2
vinculin
B
HR (long template)
% Repair
125
100
75
50
25
0
**
NTC
siBRCA2
C
HR (short template)
% Repair
125
100
75
50
25
0
***
NTC
siBRCA2

Figure S2

Figure S2. Gene silencing of BRCA2 in DLD-1 cells decreases repair of the HR reporter substrates

(A) Western blot confirming siRNA-mediated knockdown of BRCA2 in DLD-1 cells. NTC, denotes non-targeting control.
(B) After knockdown of BRCA2, DLD-1 cells display a decrease in % repair seen with the long template HR reporter.
(C) After knockdown of BRCA2, DLD-1 cells display a decrease in % repair seen with the short template HR reporter.

NanoLuc luminescence was normalised to Firefly luminescence to determine substrate repair 24 h post-transfection. In (B) and (C), % repair of siBRCA2-treated cells is expressed relative to the cells treated with a non-targeting control siRNA. Data represent mean ± SEM of 2 biological replicates, each averaging 8 technical replicates. Significance was determined by a Student’s t-test (two-tailed, unpaired).

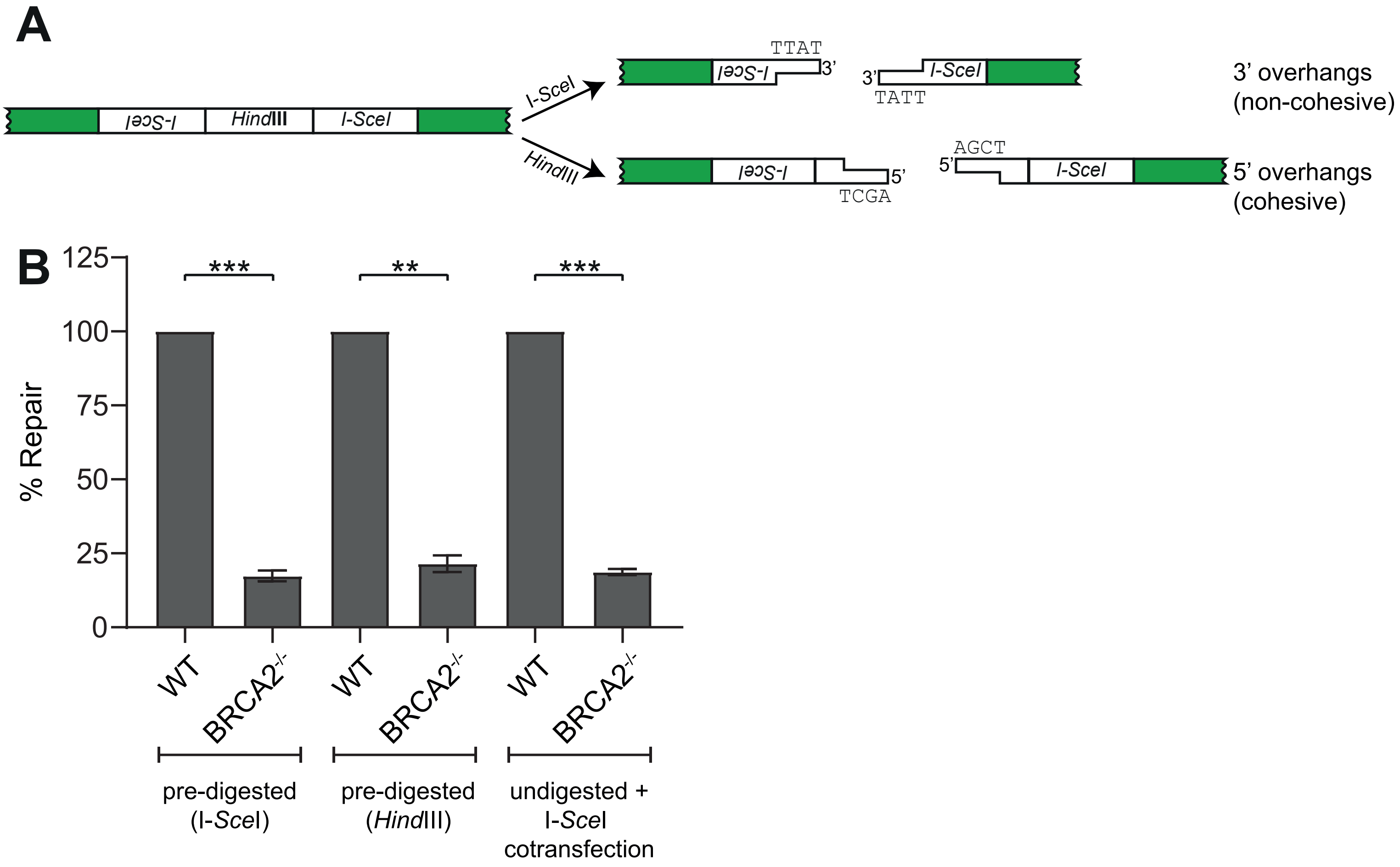
A
I-SceI
HindIII
I-SceI
I-SceI
HindIII
TTAT
I-SceI
3'
3'
I-SceI
TATT
3' overhangs
(non-cohesive)
I-SceI
5'
TCGA
AGCT
5'
I-SceI
5' overhangs
(cohesive)
B
125
100
75
50
25
0
% Repair
***
**
***
WT
BRCA2-/-
WT
BRCA2-/-
WT
BRCA2-/-
pre-digested
(I-SceI)
pre-digested
(HindIII)
undigested +
I-SceI
cotransfection

Figure S3

Figure S3. Repair of the HR reporter substrate is not impacted by method of DSB introduction.

(A) Prior to transfection, the reporter substrate can be pre-digested with restriction enzyme I-SceI, generating non-cohesive 3'-overhangs or with HindIII, generating cohesive 5'-overhangs.

(B) $BRCA2^{-/-}$ cells show defective repair of the HR reporter irrespective of the DSB generated by pre-digestion. The DSB can also be generated in cells by co-transfecting the intact reporter substrate plasmid and an I-SceI expression construct, and remains a suitable HR substrate, whose repair is inhibited in $BRCA2^{-/-}$ cells.

NanoLuc luminescence was normalised to Firefly luminescence to determine substrate repair 24 h post-transfection. % repair of $BRCA2^{-/-}$ cells is expressed relative to the parental WT cell line. Data represent mean ± SEM of 2 biological replicates, each averaging 8 technical replicates. Significance was determined by a Student's t-test (two-tailed unpaired).

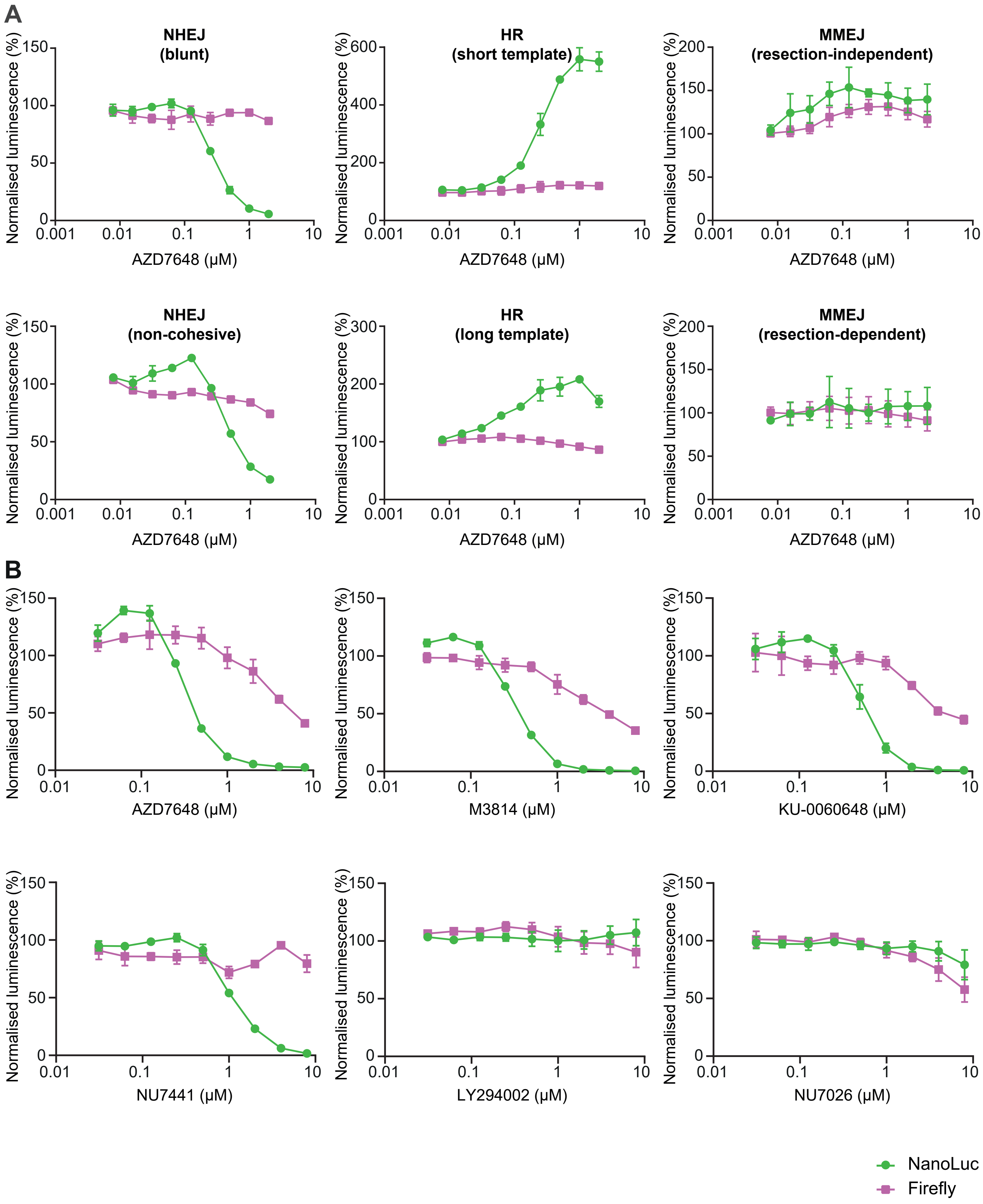
A
NHEJ
(blunt)
HR
(short template)
MMEJ
(resection-independent)
NHEJ
(non-cohesive)
HR
(long template)
MMEJ
(resection-dependent)
Normalised luminescence (%)
AZD7648 (μM)
B
M3814 (μM)
KU-0060648 (μM)
NU7441 (μM)
LY294002 (μM)
NU7026 (μM)
NanoLuc
Firefly

Figure S4

Figure S4. Pharmacological inhibition of DSB repair by DNA-PKcsi AZD7648 specifically modulates the NHEJ and HR reporter signals

(A) HEK293 cells were transfected with the indicated extrachromosomal substrates and a Firefly luciferase plasmid (transfection control) and treated with increasing concentrations of DNA-PKcs inhibitor AZD7648. The inhibition of NHEJ and up-regulation of HR are specific because of decreases and increases of the NanoLuc luminescence signals respectively, and not the Firefly control. Percentage inhibition of the luminescence signals was calculated relative to the DMSO control. % repair corresponding to these data are shown in Figure 5A-5C. Data represent mean ± SEM of 2 biological replicates, each averaging 8 technical replicates.

(B) HEK293 cells were transfected with the extrachromosomal blunt NHEJ reporter substrate and a Firefly luciferase plasmid (transfection control) and treated with increasing concentrations of indicated DNA-PKcs inhibitors. Percentage inhibition of the luminescence signals was calculated relative to the DMSO control. % repair corresponding to these data are shown in Figure 5D. The observed “increase” in repair (NanoLuc/Firefly) by NU7026 and LY294002 was driven by a reduction in the Firefly signal below the NanoLuc signal. AZD7648, M3814 and KU-0060648, but not NU7441, also displayed Firefly signal reduction at concentrations above 1 µM, suggestive of cellular toxicity, but this was less severe than the dose-dependent reduction in NanoLuc. Data represent mean ± SEM of 2 biological replicates, each averaging 4 technical replicates.

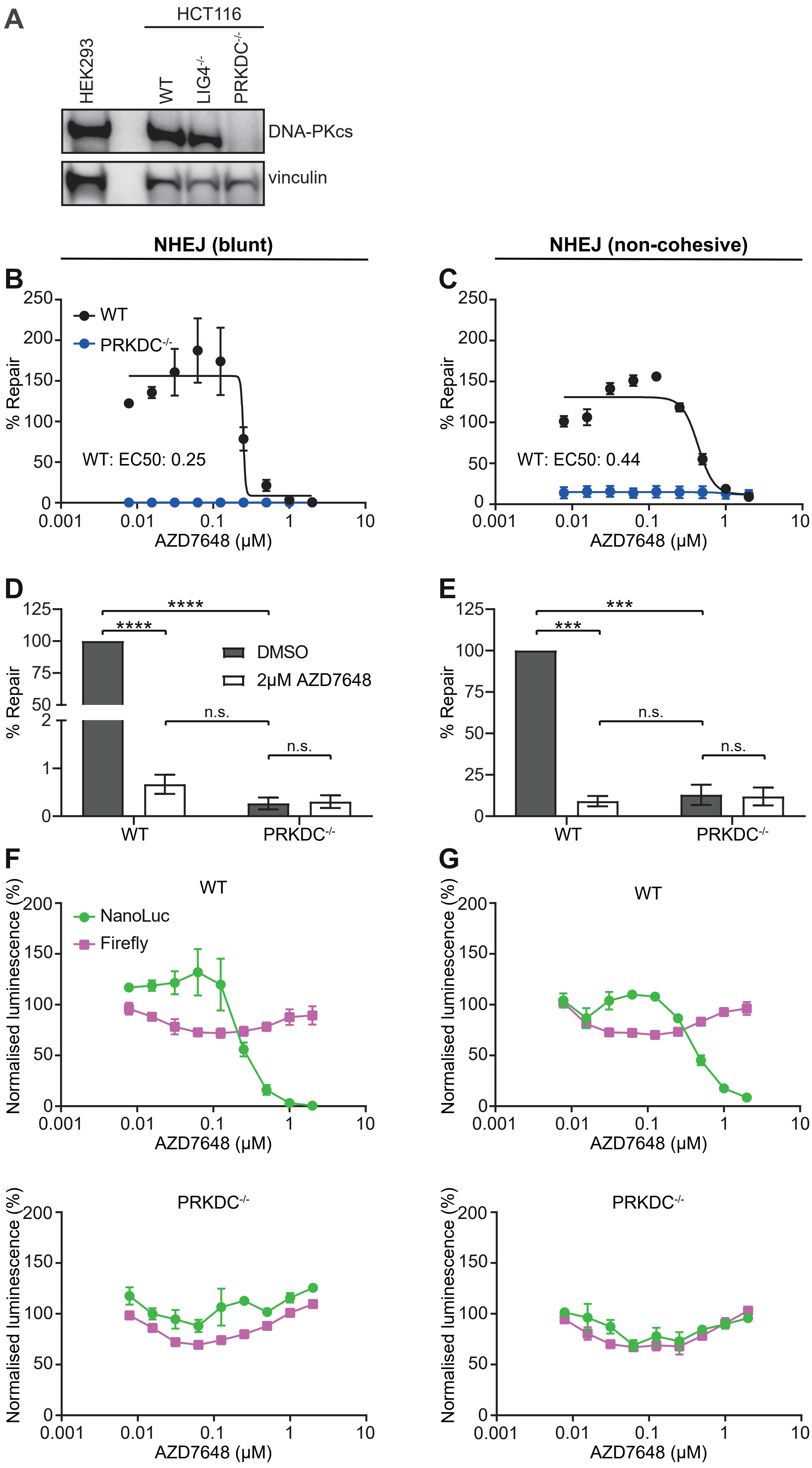
A
HCT116
HEK293
WT
LIG4-/-
PRKDC-/-
DNA-PKcs
vinculin
NHEJ (blunt)
NHEJ (non-cohesive)
B
C
WT
PRKDC-/-
% Repair
WT: EC50: 0.25
WT: EC50: 0.44
AZD7648 (μM)
D
E
DMSO
2μM AZD7648
****
***
n.s.
F
G
NanoLuc
Firefly
Normalised luminescence (%)

Figure S5

Figure S5. Pharmacological inhibition of NHEJ reporter signal by DNA-PKcsi AZD7648 in WT and DNA-PKcs knockout cells

(A) Western blot of isogenic HCT116 cell lines confirming loss of DNA-PKcs protein in PRKDC$^{-/-}$ cells. HEK293 cells are shown for comparison.

HCT116 cells (WT and PRKDC$^{-/-}$) were transfected with the indicated extrachromosomal substrates (Blunt NHEJ in (B), (D) and (F), and non-cohesive NHEJ in (C), (E) and (G)) and a Firefly luciferase plasmid (transfection control) and treated with increasing concentrations of DNA-PKcs inhibitor AZD7648. NanoLuc luminescence was normalised to Firefly luminescence to determine substrate repair 16h post-transfection. Percentage inhibition was calculated relative to the DMSO control.

(B) and (C) Inhibition of DNA-PKcs by AZD7648 caused a dose-dependent suppression of NHEJ in WT but not PRKDC$^{-/-}$ cells.
(D) and (E) % repair was normalised to levels in WT cells after DMSO treatment. In the absence of AZD7648, PRKDC$^{-/-}$ cells had a significant defect in NHEJ compared to WT cells. 2 µM AZD7648 significantly inhibited NHEJ in WT cells to levels in PRKDC$^{-/-}$ cells. No additional effect of AZD7648 was observed in PRKDC$^{-/-}$ cells.
(F) and (G) Percentage inhibition of the individual luminescence signals in (B) and (E) was calculated relative to the DMSO control. The dose-dependent inhibition of NHEJ in WT cells in (B) and (E) specifically resulted from the NanoLuc luminescence signals, not the Firefly control (left panel). In PRKDC$^{-/-}$ cells, no inhibition of NanoLuc luminescence was observed (right panel).

Data represent mean ± SEM of 2 biological replicates, each averaging 8 technical replicates. Significance was determined by a Two-Way Analysis of Variance (ANOVA).

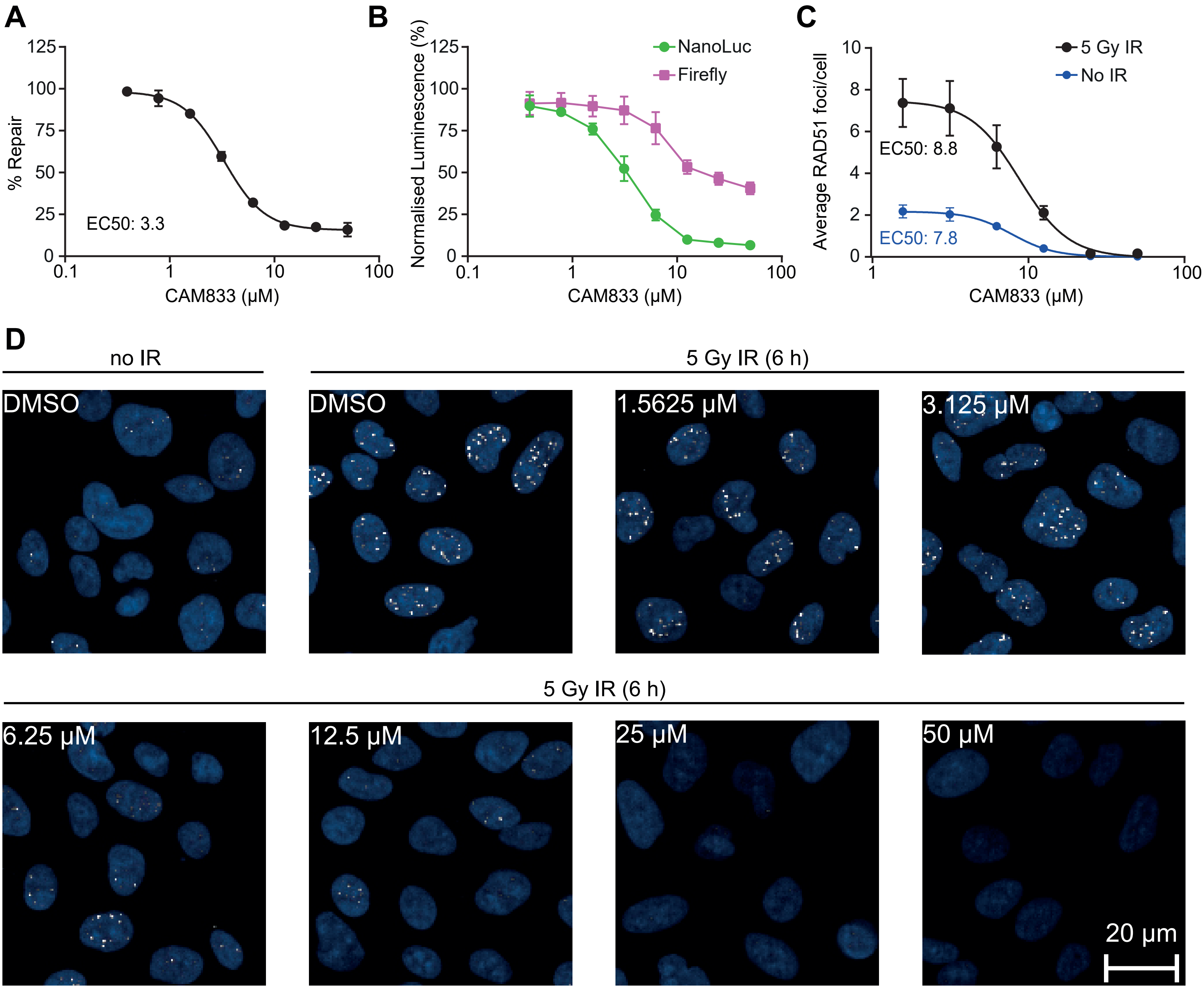
A
125
100
75
50
25
0
% Repair
EC50: 3.3
0.1
1
10
100
CAM833 (µM)
B
Normalised Luminescence (%)
NanoLuc
Firefly
CAM833 (µM)
C
Average RAD51 foci/cell
5 Gy IR
No IR
EC50: 8.8
EC50: 7.8
CAM833 (µM)
D
no IR
5 Gy IR (6 h)
DMSO
DMSO
1.5625 µM
3.125 µM
5 Gy IR (6 h)
6.25 µM
12.5 µM
25 µM
50 µM
20 µm

Figure S6

Figure S6. Pharmacological inhibition of HR by RAD51i CAM833

(A) HEK293 cells were transfected with the extrachromosomal HR substrate and a Firefly luciferase plasmid (transfection control) and treated with increasing concentrations of RAD51i CAM833. NanoLuc luminescence was normalised to Firefly luminescence to determine substrate repair 16 h post-transfection. Percentage inhibition was calculated relative to the DMSO control. Data represent mean ± SEM of 2 biological replicates, each averaging 4 technical replicates.

(B) The inhibition of HR by CAM833 is driven by modulation of the NanoLuc reporter substrate luminescence signal. However, at concentrations above 12 µM, the Firefly luminescence signal is also impacted indicating potential cellular toxicity. Percentage inhibition of the luminescence signals was calculated relative to the DMSO control.

(C) CAM833 suppresses the formation of IR-induced RAD51 foci.

(D) Representative immunofluorescence images from (C) at indicated concentrations of CAM833.

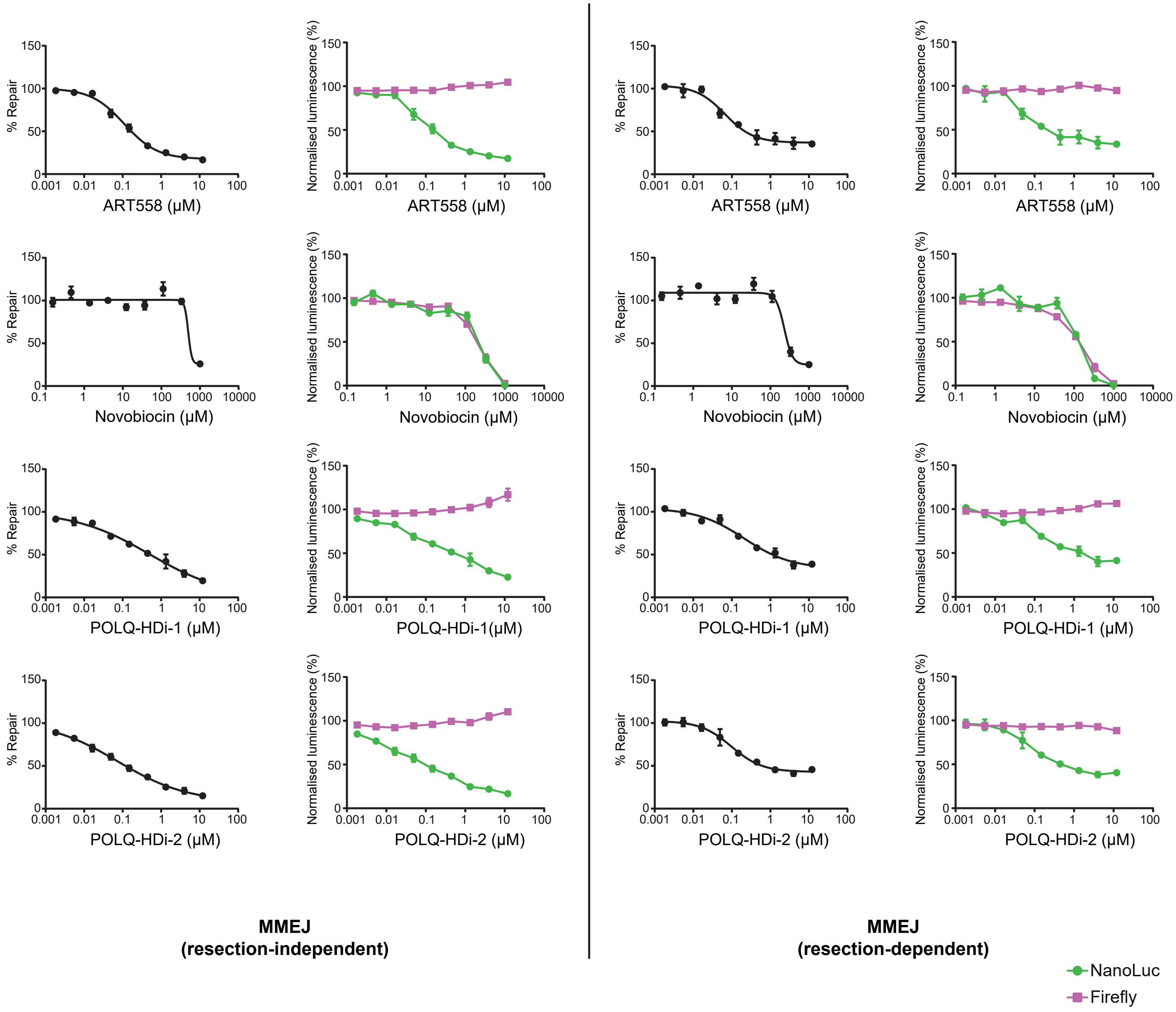
% Repair
Normalised luminescence (%)
ART558 (µM)
Novobiocin (µM)
POLQ-HDi-1 (µM)
POLQ-HDi-1(µM)
POLQ-HDi-2 (µM)
MMEJ
(resection-independent)
MMEJ
(resection-dependent)
NanoLuc
Firefly

Figure S7

Figure S7. Evaluation of Polθ helicase inhibitors in MMEJ assays
HEK293 cells were transfected with the indicated extrachromosomal substrates and a Firefly luciferase plasmid (transfection control) and treated with increasing concentrations of an active inhibitor of Polθ polymerase (ART558), or reported inhibitors of the Polθ helicase (Novobiocin, POLθ-HDi1 or POLθ-HDi2). NanoLuc luminescence was normalised to Firefly luminescence to determine substrate repair 24 h post-transfection (black lines). Percentage inhibition was calculated relative to the DMSO control. Percentage inhibition of the individual NanoLuc and Firefly luminescence signals are also shown (green and magenta lines).

ART558 caused a dose-dependent inhibition of both resection-independent and -dependent MMEJ (EC50 0.11 µM and 0.066 µM, respectively). Novobiocin did not have any effect on MMEJ repair at doses reported to inhibit Polθ (45) and up to 1 mM without concomitant effects on the control Firefly signal. POLθ-HDi1 and POLθ-HDi2 did specifically inhibit MMEJ. EC50s were estimated at 0.51 µM and 0.075 µM (resection-independent MMEJ) and 0.21 µM and 0.097 µM (resection dependent MMEJ), respectively, but robust plateaus could not be determined to accurately define these values.

Data represent mean ± SEM of 2 biological replicates, each averaging 4 technical replicates.

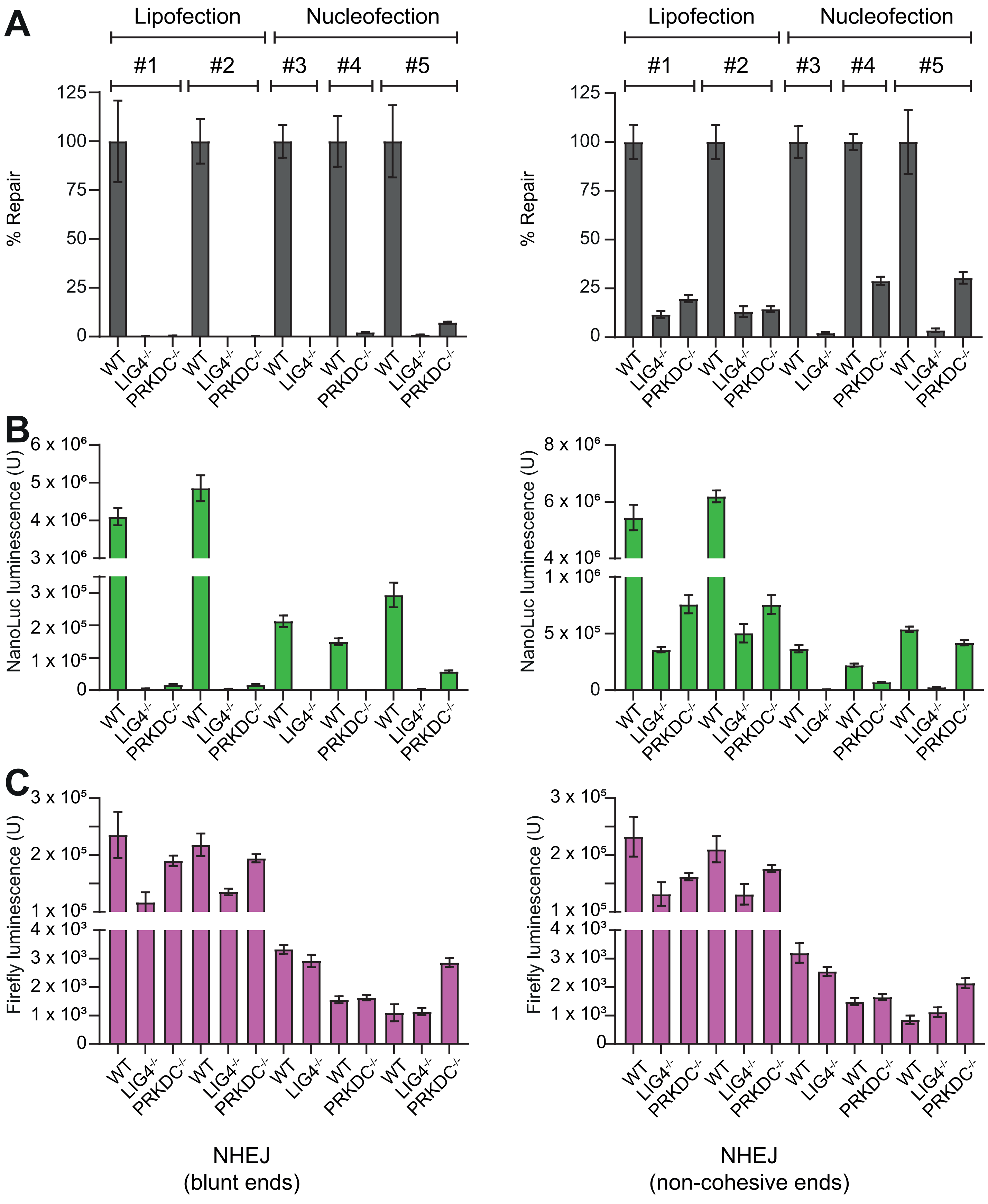
A
Lipofection
Nucleofection
#1
#2
#3
#4
#5
% Repair
125
100
75
50
25
0
WT
LIG4-/-
PRKDC-/-
B
NanoLuc luminescence (U)
6 x 10⁶
5 x 10⁶
4 x 10⁶
3 x 10⁶
3 x 10⁵
2 x 10⁵
1 x 10⁵
8 x 10⁶
1 x 10⁶
5 x 10⁵
C
Firefly luminescence (U)
3 x 10⁵
2 x 10⁵
1 x 10⁵
4 x 10³
3 x 10³
2 x 10³
1 x 10³
NHEJ
(blunt ends)
NHEJ
(non-cohesive ends)

Figure S8

Figure S8. Extrachromosomal substrates can be transfected into cells by lipofection or nucleofection

LIG4$^{-/-}$ and PRKDC$^{-/-}$ HCT116 cells were transfected with two NHEJ reporter substrates using lipofection (runs #1 and #2) or nucleofection (runs #3, #4 and #5).

(A) NanoLuc luminescence was normalised to Firefly luminescence to determine substrate repair 24 h post-transfection. Consistent repair levels were observed between independent experiments. % repair of knockout cells is expressed relative to their respective parental WT cell line.

(B) NanoLuc signals alone from experiments in (A).

(C) Firefly signals alone from experiments in (A). The co-transfected Firefly plasmid was expressed from pGL4.53[luc2/PGK].

Data represent mean ± SEM of 2 biological replicates, each averaging 8 technical replicates.

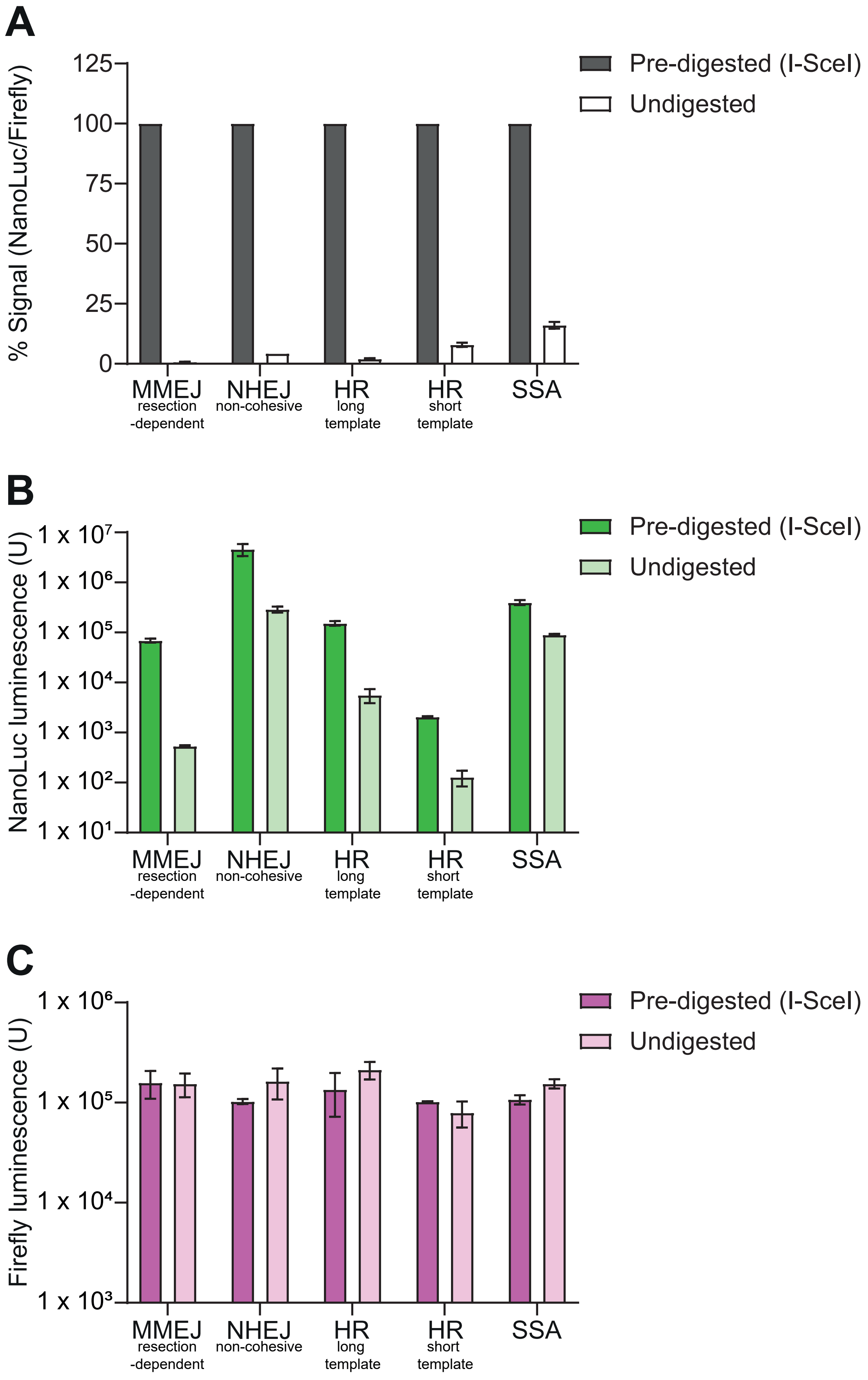
A
% Signal (NanoLuc/Firefly)
125
100
75
50
25
0
Pre-digested (I-SceI)
Undigested
MMEJ
resection
-dependent
NHEJ
non-cohesive
HR
long
template
HR
short
template
SSA
B
NanoLuc luminescence (U)
1 x 10⁷
1 x 10⁶
1 x 10⁵
1 x 10⁴
1 x 10³
1 x 10²
1 x 10¹
Pre-digested (I-SceI)
Undigested
MMEJ
resection
-dependent
NHEJ
non-cohesive
HR
long
template
HR
short
template
SSA
C
Firefly luminescence (U)
1 x 10⁶
1 x 10⁵
1 x 10⁴
1 x 10³
Pre-digested (I-SceI)
Undigested
MMEJ
resection
-dependent
NHEJ
non-cohesive
HR
long
template
HR
short
template
SSA

Figure S9

Figure S9. I-SceI pre-digested reporter substrates are required to generate NanoLuc signals upon transfection

HEK293 cells were transfected with resection-dependent MMEJ, NHEJ, HR and SSA reporters after I-SceI pre-digestion of the parental plasmid, mimicking a double strand break, or alternatively uncut, alongside a Firefly control.

(A) NanoLuc luminescence was normalised to Firefly luminescence to determine substrate repair 24 h post-transfection. The responses of uncut plasmids were normalised to cut plasmids. Digested plasmids generated robust repair signals.
(B) NanoLuc signals alone show that I-SceI pre-digested substrates generate NanoLuc signals.
(C) The co-transfected Firefly plasmid signal, acting as a control, is stable whether the reporter substrate is pre-digested or uncut.

Data represent mean ± SEM of 2 biological replicates, each averaging 8 technical replicates.

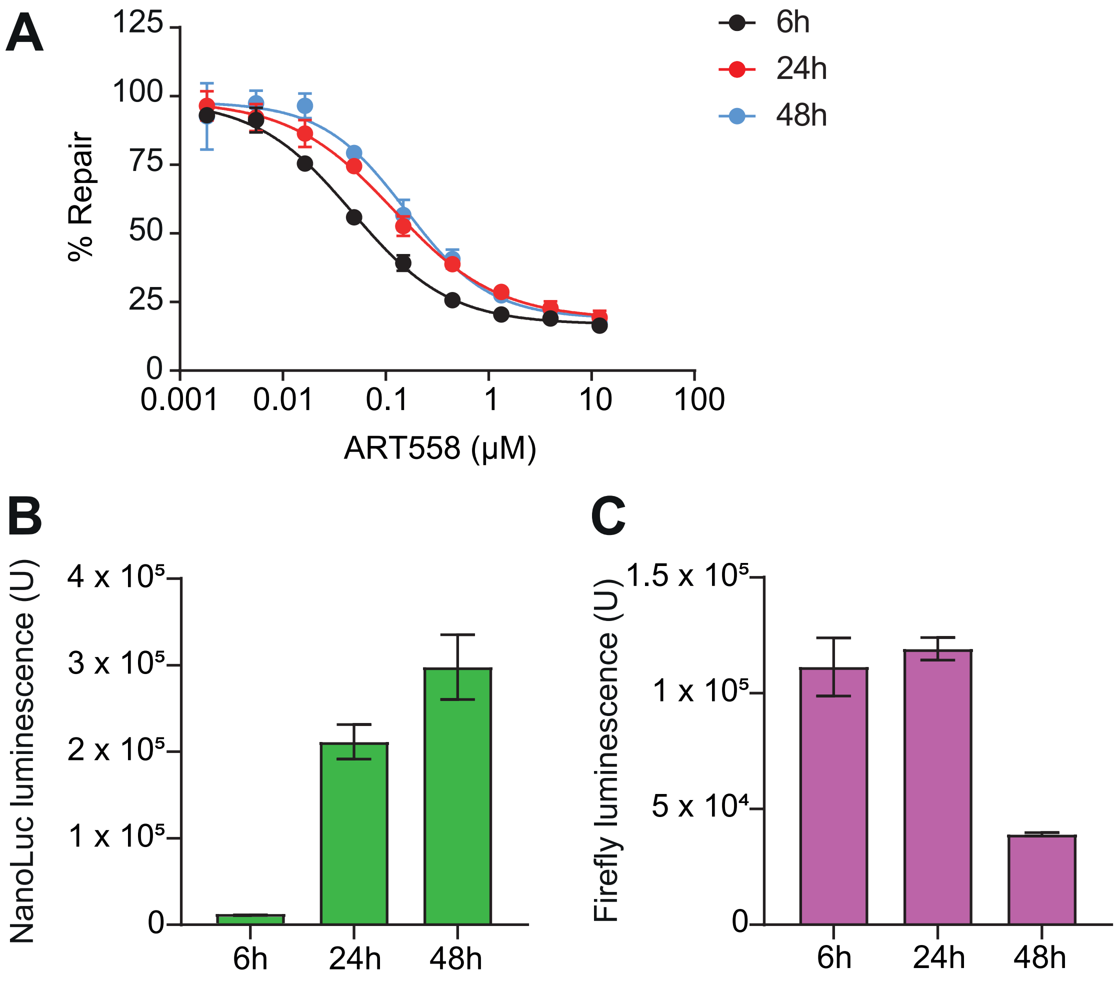
A
125
100
75
50
25
0
% Repair
0.001
0.01
0.1
1
10
100
ART558 (µM)
6h
24h
48h
B
NanoLuc luminescence (U)
4 x 10⁵
3 x 10⁵
2 x 10⁵
1 x 10⁵
0
6h
24h
48h
C
Firefly luminescence (U)
1.5 x 10⁵
1 x 10⁵
5 x 10⁴
0
6h
24h
48h

Figure S10

Figure S10. Resection-independent MMEJ assays performed at multiple timepoints.
HEK293 cells were transfected with the resection-independent MMEJ substrates and a Firefly luciferase plasmid (transfection control) and treated with increasing concentrations of an active inhibitor of Polθ (ART558). NanoLuc luminescence was normalised to Firefly luminescence to determine substrate repair at 6 h, 24 h and 48 h post-transfection. Percentage inhibition was calculated relative to the DMSO control.

(A) ART558 caused a dose-dependent inhibition of MMEJ at each timepoint and EC50s were within approximately three-fold (6 h: 0.047 µM, 24 h: 0.119 µM, 48 h: 0.157 µM).
(B) NanoLuc signals are detected at indicated timepoints.
(C) The co-transfected Firefly plasmid signal, acting as a control, is detected at indicated timepoints.

Data represent mean ± SEM of 2 biological replicates, each averaging 4 technical replicates.

SUPPLEMENTARY TABLES

Supplementary Table 1: Oligonucleotides

Sequence (5'-3')	Supplier	Purification	Function
5'[Phos]CATTCTGGCGTAAGGCCGCGACT	Sigma	PAGE	PEST excision
5'[Phos]CTAGAGTCGCGGCCTTACGCCAGAATG	Sigma	PAGE	PEST excision
5'[Phos]TCGAGGACTTGGTCCAGGTTGTAGCCGGCTG TCTGTCGCCAGTCCCCAACGAAATCTTCGAGTGTGA AGACCAT	Sigma	PAGE	Long left cap for resection-independent MMEJ
5'[Phos]GCCGGCTACAACCTGGACCAAGTCC	Sigma	PAGE	Short left cap for resection-independent MMEJ
5'[Phos]AGCTTTATTGCGGTAGTTTATCACAGTTAAAT TGCTAACGCAGTCAGTGGGCCTCGGCGGCCAAGCTA GGCAATCCGGTACTGTTGGTAAAGCCACCATGG	Sigma	PAGE	Long right cap for resection-independent MMEJ
5'[Phos]CGAGGCCCACTGACTGCGTTAGCAATTTAAC TGTGATAAACTACCGCAATAA	Sigma	PAGE	Short right cap for resection-independent MMEJ

Supplementary Table 2: Cell lines

Name	Supplier	Product Code	Media
DLD-1	Horizon Discovery	HD PAR-008	RPMI1640 (PAN Biotech, P04-04510) + 10% FBS (PAN Biotech, P30-3306)
DLD-1 BRCA2-/-	Horizon Discovery	HD 105-007	RPMI1640 + 10% FBS
HCT116 PAR-082	Horizon Discovery	HD PAR-082	RPMI1640 + 10% FBS
HCT116 LIG4-/-	Horizon Discovery	HD R02-063	RPMI1640 + 10% FBS
HCT116 PRKDC-/-	Horizon Discovery	HD R02-049	RPMI1640 + 10% FBS
HCT116 XRCC4-/-	Horizon Discovery	HD R02-076	RPMI1640 + 10% FBS
HCT116 XLF-/-	Horizon Discovery	HD R02-068	RPMI1640 + 10% FBS
eHAP1 Parental	Boulton lab	n/a	IMDM (Gibco, 12440-053) + 10% Tetracycline-free FBS (Pan Biotech, P30-3602)
eHAP1 POLQ(-)	Boulton lab	n/a	IMDM + 10% Tetracycline-free FBS
HEK293	ATCC	CRL-1573	MEM Eagle (PAN Biotech, P04-08056) + 10% FBS
U-2 OS Parental	ATCC	HTB-96	McCoy's 5A Medium (PAN Biotech, P04-06500) + 10% FBS
U-2 OS POLQ KO clone C2	Synthego	n/a	McCoy's 5A Medium + 10% FBS
U-2 OS Flp-In T-REx GFP-POLQ	Artios	n/a	DMEM (high glucose) (PAN Biotech, P04-04510) + 10% Tetracycline-free FBS + 200 µg/mL Hygromycin B + 8 µg/mL Blasticidin S

Supplementary Table 3: siRNA

siRNA	Target	Supplier	Product code
ON-TARGETplus Human BRCA1 siRNA SMARTpool	BRCA1	Horizon Discovery/Dharmacon	L-003461-00-0005
ON-TARGETplus Human BRCA2 siRNA SMARTpool	BRCA2	Horizon Discovery/Dharmacon	L-003462-00-0005
ON-TARGETplus Human PALB2 siRNA SMARTpool	PALB2	Horizon Discovery/Dharmacon	L-012928-01-0005
ON-TARGETplus Human RAD51 siRNA SMARTpool	RAD51	Horizon Discovery/Dharmacon	L-003530-00-0005
ON-TARGETplus Human RAD52 siRNA SMARTpool	RAD52	Horizon Discovery/Dharmacon	L-011760-00-0005
ON-TARGETplus Non-targeting Control Pool	n/a	Horizon Discovery/Dharmacon	D-001810-10-05

Supplementary Table 4: Antibodies

Primary antibodies	Supplier	Product Code	Dilution	Application
BRCA1	Santa Cruz Biotechnology	sc-6954	1:500	WB
BRCA2	Sigma-Aldrich	OP95	1:1000	WB
GAPDH	Cell Signaling Technology	3683S	1:5000	WB
GFP	Evrogen	AB011	1:1000	WB
LIG4	Abcam	ab193353	1:1000	WB
PALB2	Bethyl laboratories	A301-246A	1:1000	WB
Polθ	Artios	n/a	1:250	WB
PRKDC	Cell Signaling Technology	4602S	1:1000	WB
RAD51	Millipore	ABE257	1:5000	WB, IF
RAD52	Abcam	ab124971	1:1000	WB
Vinculin	Santa Cruz Biotechnology	sc-73614	1:2000	WB
XLF	Bethyl laboratories	A300-730A	1:1000	WB
XRCC4	Santa Cruz Biotechnology	sc-271087	1:1000	WB
α-tubulin	Abcam	ab7291	1:5000	WB

Secondary antibodies	Supplier	Product Code	Dilution	Application
Anti-Rabbit Alexa Fluor 488	Invitrogen	A11034	1:2000	IF
Goat anti-Mouse IgG (H+L) Secondary Antibody, HRP	Invitrogen	31430	1:5000	WB
Goat anti-Rabbit IgG (H+L) Secondary Antibody, HRP	Invitrogen	31460	1:5000	WB
IRDye 800CW Goat anti-Mouse IgG Secondary Antibody	LI-COR	926-32210	1:15000	WB
IRDye 800CW Goat anti-Rabbit IgG Secondary Antibody	LI-COR	926-32211	1:15000	WB
IRDye 680RD Goat anti-Mouse IgG Secondary Antibody	LI-COR	926-68070	1:15000	WB
IRDye 680RD Goat anti-Rabbit IgG Secondary Antibody	LI-COR	926-68071	1:15000	WB

Supplementary Table 5: Compounds

Name	Supplier	Product Code
ART558 (Polθi)	Artios	n/a
ART615 (Polθi)	Artios	n/a
AZD7648 (DNA-PKi)	SelleckChem	S8843
Novobiocin sodium	SelleckChem	S2492
CAM833 (RAD51i)	Bio-Techne Ltd	7457/10
M3814 (DNA-PKi)	ChemieTek	CT-M3814
KU-0060648 (DNA-PKi)	SelleckChem	S8045
NU7441 (DNA-PKi)	SelleckChem	S2638
LY294002 (DNA-PKi)	SelleckChem	S1105
NU7026 (DNA-PKi)	MedChemexpress	HY-15719
POLQ-HDi1	Synthesised at Pharmaron	Ex. 177 from WO 2020/243459
POLQ-HDi2	Synthesised at Pharmaron	Ex. 224 from WO 2020/243459

Supplementary Table 6: Software

Software	Version	Supplier
GraphPad Prism	9.4.0	GraphPad
Excel	2303 (build 16.0.16227.20202)	Microsoft
Adobe illustrator	27.4.1	Adobe
Harmony	4.9	Perkin Elmer
Empiria Studio	2.2	LI-COR

The graphical abstract available at NAR online was created using BioRender.com.

Supplementary Table 7: Comparison of DSBR reporter systems

		Extrachromosomal NanoLuc-based reporters	Chromosomally integrated fluorescence-based reporters
Assay format	**Reporter gene**	Nanoluciferase: Low molecular weight, fast maturing reporter with high stability and sensitivity (25).	Standard fluorophores such as GFP are well established research tools and can be easily detected by imaging and flow cytometry.
	**DSB induction**	DSB is generated in reporter prior to transfection by restriction digest and/or standard molecular biology. Gel-based assessment ensures high efficiency. Where DSB generation is dependent on I-SceI, intact reporter plasmid can be co-transfected with I-SceI.	DSB is introduced in genomically integrated reporter by transient transfection or induction of DSB-inducing enzyme.
	**Assay run time**	Luminescence can be measured in <24 h.	Fluorescence can be measured in 48-96 h.
	**Reporter signal detection**	Plate-based assay using in situ addition of luminescence detection reagents. Workflow suitable for medium-high throughput format using multidispenser/liquid handler.	Flow cytometry: samples harvested, washed and resuspended prior to flow cytometric analysis. For high throughput format, samples may be transferred to multiwell plate (51). Alternatively, plate-based imaging is possible after sample washing, fixation and DNA co-stain (50).
	**Equipment required**	Plate reader equipped with luminescence detection, acquisition ~5 mins per 96-well plate.	Flow cytometer equipped with appropriate fluorescence channels e.g. GFP/RFP, ~1-2 mins per sample. High throughput format requires autosampler connected to flow cytometer. Alternatively, may use high content microscopy.
DSBR analysis	**DSB functionalisation**	Engineered using standard in vitro molecular biology techniques such as restriction digest and annealing of structured/modified oligonucleotides onto core reporter substrate prior to transfection.	Engineered by Cas9 variants or guide spacing in reporter (52).
	**Flexibility of model systems**	Any cell line amenable to transient lipofection or nucleofection of reporter substrate. Reporter can be used in isogenic cell lines and/or upon siRNA-mediated depletion of gene of interest in any transfectable cell line.	Stable cell line containing reporter construct. Cell line editing is required to introduce reporter construct. Knockout or knockdown of the gene of interest is restricted to cell line in which reporter has been integrated.
	**DSBR context**	Reporter is extrachromosomal and is therefore not in a physiological context. This may limit the sensitivity of the reporter to factors that are not required for chromatin remodelling and other regulatory factors. Reporter substrates can be available at high levels, potentially sequestering repair factors and functioning outside of cell cycle control.	Reporter is chromosomally integrated (ideally single copy) and DSB is therefore in a native, physiological, chromatinised context. As reporter is stably integrated, positive and negative influences from genomic context of specific integration site may influence the repair (54).
	**DNA repair event sequencing**	Feasible based on PCR amplicon NGS-sequencing e.g. similar to (55).	Established in literature and allows sensitivity analysis of sequence scars at repaired breaks (46, 56).
	**Titratable**	Demonstrable titratability with DDR inhibitors suitable for assessment of cellular potency and correlation.	Pharmacological sensitivity has been shown using flow cytometry or a sequencing readout to measure repair events (46, 53).
	**Multiplexing**	This study describes DSBR reporters assessing MMEJ, NHEJ, HR and SSA that can be used in isolation.	Comprehensive range of DSBR reporter systems available, reviewed in (18), and potential for simultaneous assessment of multiple pathways with a single reporter e.g. TLR (51) and DSB-Spectrum (53).

SUPPLEMENTARY REFERENCES

54. Schep,R., Brinkman,E.K., Leemans,C., Vergara,X., van der Weide,R.H., Morris,B., van Schaik,T., Manzo,S.G., Peric-Hupkes,D., van den Berg,J., *et al.* (2021) Impact of chromatin context on Cas9-induced DNA double-strand break repair pathway balance. *Mol. Cell*, **81**, 2216-2230.e10.
55. de Muinck,E.J., Trosvik,P., Gilfillan,G.D., Hov,J.R. and Sundaram,A.Y.M. (2017) A novel ultra high-throughput 16S rRNA gene amplicon sequencing library preparation method for the Illumina HiSeq platform. *Microbiome*, **5**, 68.
56. Taheri-Ghahfarokhi,A., Taylor,B.J.M., Nitsch,R., Lundin,A., Cavallo,A.-L., Madeyski-Bengtson,K., Karlsson,F., Clausen,M., Hicks,R., Mayr,L.M., *et al.* (2018) Decoding non-random mutational signatures at Cas9 targeted sites. *Nucleic Acids Res.*, **46**, 8417–8434.
